# Underestimated role of macromolecular crowding in bioengineered *in vitro* models of health and diseases

**DOI:** 10.1016/j.mtbio.2025.101772

**Published:** 2025-04-17

**Authors:** Jérémy Lagrange, Gabrielle Van De Velde, Patrick Lacolley, Véronique Regnault, Rümeyza Bascetin

**Affiliations:** a Université de Lorraine, Inserm, DCAC, F-54000, Nancy, France; b Université de Lorraine, CHRU-Nancy, Inserm, IHU INFINY, F-54000, Nancy, France

**Keywords:** Macromolecular crowding, Biomimetic, Microenvironment, Vascular biology, Cancer, Neurodegenerative disorders

## Abstract

Macromolecular crowding (MMC) is a ubiquitous phenomenon in biological systems that is largely overlooked in bioengineered *in vitro* cellular models. This comprehensive review examines the significant impact of both intracellular and extracellular MMC on cellular and molecular processes under physiological and pathological conditions. By synthesizing current knowledge and identifying critical gaps in our understanding of MMC, this review highlights the need to incorporate crowding into the development of *in vitro* models for studying health and diseases, as well as for drug discovery platforms. The pervasive nature of MMC in biological systems underscores its potential importance in various physiological and pathological processes, including protein aggregation disorders, cancer, and vascular diseases. Recognizing the ubiquitous influence of MMC could open new avenues for therapeutic interventions and deepen our understanding of fundamental biological processes.

## Graphical abstract

**Figure:**
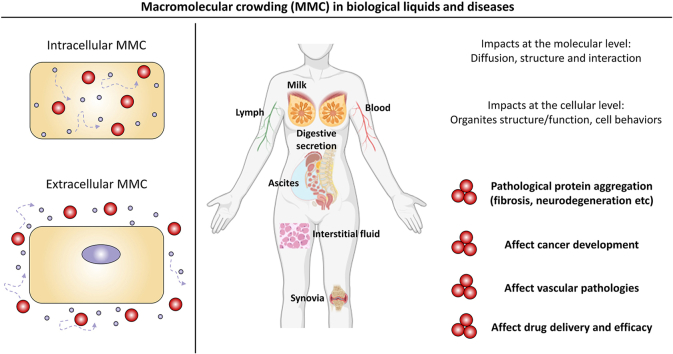

**Table:** Glossary

**Ascites**	Accumulation of fluid in the abdominal cavity. It generally originates from blood and lymph effusion. It is often observed in advanced stages of certain cancers and especially ovarian cancer.
**Anterogradetransport**	it directs the movement of organelles from the cell's center to its periphery, including the cell membrane and specialized compartments. This transport system utilizes microtubules as tracks, with kinesin motor proteins propelling the cargo. The process facilitates the movement of diverse cellular components, such as synaptic and secretory vesicles, membrane lipids, and proteins intended for cell surface expression or secretion.
**Chloramphenicol**	It is a broad-spectrum antibiotic that inhibits protein synthesis. It specifically inhibits the translation of mitochondrial mRNA, leading to a decrease in the synthesis of mitochondrial-encoded proteins.
**Doxorubicin**	It is a cytotoxic cancer drug inhibiting topoisomerase II and thus cell growth. It is used in several cancer treatments including ovarian cancer.
**FapC**	It serves as the principal protein component in the formation of amyloid fibrils, which play a crucial role in reinforcing bacterial biofilms and enhancing their resilience against dehydration.
**Fibrosis**	It is a thickening and scarring of connective tissue characterized by misfolding of proteins and, by stiffening and decreasing elasticity of the tissue.
**Fractional volume occupancy**	It quantifies the volume fraction of macromolecules in a solution, representing the proportion of space they occupy relative to the total solution volume.
**Genotoxicity**	It is a toxicity on cells induced by damage on DNA by an active compound.
**Hydrodynamic radius**	It represents the size of a molecule in solution, accounting for its shape and associated water molecules. It is equivalent to the radius of a theoretical hard sphere that would exhibit the same diffusion rate as the molecule under consideration.
**Interstitial fluid**	Fluid found in the space around cells in tissues. It is defined as the fluid circulating in tissues outside of the biological fluid network (vascular network, lymphatic network, cerebrospinal liquid etc).
**Intrinsically disordered proteins**	They are proteins characterized by their inherent lack of a stable, well-defined three-dimensional structure under physiological conditions. These proteins exist in a dynamic state of structural flexibility, adopting an ensemble of rapidly interconverting conformations rather than a single, fixed tertiary structure.
**Liquid-liquid phaseseparation (LLPS)**	It is phenomenon occurring when a homogeneous mixture containing multiple components spontaneously separates into two distinct liquid phases, each with varying concentrations of components.
**Marfan syndrome**	It is a pathology where tissue elasticity is compromised due to a pathogenic variant of fibrillin-1 protein. Fibrillin-1 deposition is decreased.
**NPM1**	Nucleophosmin 1 is a multifunctional protein that predominantly resides in the nucleolus but possesses the ability to move dynamically between the nucleus and the cytoplasm. Like SURF6, it plays crucial roles in the production of ribosomes and the regulation of cell division processes.
**Polydispersity (PDI)**	It quantifies the breadth of a polymer's molecular weight distribution. It is calculated as the ratio of weight-average molecular weight to number-average molecular weight. A higher PDI value indicates a broader range of molecular weights within the polymer sample.
**Quinacrine**	It is an antiprotozoal agent killing or inhibiting the growth of protozoa.
**Retrograde transport**	It directs the movement of organelles from the cell's periphery, such as the cell membrane or specialized compartments, towards its center. This process employs microtubules as tracks, with dynein motor proteins serving as the driving force for movement. The transport system facilitates the movement of diverse cellular components, including endocytosed materials, signaling molecules, and recycled proteins and lipids, from the cell's outer regions to its interior.
**SB216763**	It is an ATP-competitive inhibitor that effectively targets glycogen synthase kinase-3 (GSK-3) with high specificity. It stimulates glycogen synthesis and induces β-catenin-dependent gene transcription. It is commonly used in research to study the role of GSK-3 in various cellular processes and signaling pathways including reducing pulmonary inflammation and fibrosis in mouse models.
**SK-HEP1**	It is an endothelial cell line derived from hepatic adenocarcinoma. It was first isolated and established from ascitic fluid of a patient with hepatic carcinoma. It is used as a hepatocellular carcinoma cell but it is also used as a cell model for liver sinusoidal endothelial cells as they present endothelial markers, functions and tubular formation.
**SURF6**	Surfeit locus protein 6, a highly conserved protein found in the nucleolus, exhibits a robust ability to bind nucleic acids under laboratory conditions, demonstrating a particular preference for RNA over DNA. This protein plays crucial roles in the production of ribosomes and the regulation of cell division processes.

## 1 Introduction

Despite the high density of macromolecules in biological fluids both inside and outside cells, systematic studies on the impact of crowding on cells, tissues and organs physiology remain scarce. Macromolecular crowding (MMC) refers to the presence of a high density of macromolecules such as proteins, nucleic acids, polysaccharides, and lipids in biological fluids inside cells and in the extracellular environment like interstitial liquid, blood or ascites.

The ubiquitous nature of MMC in biological systems underscores its potential significance in both physiological and pathological processes. Investigating its effects on molecular interactions, protein folding, and cellular signaling pathways could provide valuable insights into its role in maintaining cellular function and its implications for diseases progression. Moreover, integrating MMC into the development of *in vitro* cell-free and cell-based models, as well as drug discovery platforms, could lead to innovative therapeutic strategies and enhance our understanding of biological processes in complex intracellular and extracellular environments that more closely resemble *in vivo* conditions.

This review aims to highlight the potential of MMC in modeling health and diseases *in vitro* by replicating physiological culture conditions or creating crowded environments in cell-free systems. This review synthesizes current knowledge on MMC and identifies critical gaps in our understanding, emphasizing the need for further research in this field.

This review provides fundamental background to understand the role and effects of MMC at the molecular and cellular levels, both inside and outside the cell. It outlines strategies for engineering MMC-based *in vitro* cell-free and cell-based models and, offers an overview of existing literature on MMC models used to study health and diseases, particularly in the contexts of pathological protein polymerization, osteoarthritis, infection-related biofilm formation, tumor development, and vascular biology. Additionally, it presents the "scar-in-a-jar" model designed to mimic fibrosis and evaluate drug efficacy. Future research directions are also proposed at the end of the article.

## 2 The crowded nature of cytoplasm and biological fluids

A crucial yet often underappreciated feature of biological systems is the high density of macromolecules within a restricted volume. These environments are referred to as "crowded" or "volume-occupied" rather than simply "concentrated" [1]. MMC is a well-established phenomenon within the cell interior and is also observed in the extracellular environment [1,2].

The density of macromolecules inside cells, including the nucleus, ranges from 75 to 400 mg/mL [[3], [4], [5]]. Biological fluids such as interstitial fluid, blood, and ascites contain between 20 and 80 mg/mL of macromolecules, with some instances reaching even higher concentrations [[2], [6], [7]]. Although the exact concentration of each macromolecule is not fully characterized, the high density and diversity of macromolecules in the extracellular matrix (ECM) surrounding cells in both soft and hard tissues are well recognized [8].

This implies that, *in vivo*, molecules and cells exist in continuously crowded microenvironments, whereas *in vitro*, biochemists and biologists often study molecules and cells in diluted cell-free and cell-based media where crowding is negligible [9]. For instance, culture media are highly diluted compared to physiological conditions, containing only 0.5 %–10 % of the total serum macromolecules concentration, which is at least ten time less crowded than blood for endothelial cell culture. While the effects of MMC at the molecular level are well documented, its impact at the cellular and tissue levels remains less understood—although growing interest in this field is beginning to change this trend.

## 3 Macromolecular crowding fundamentals at the molecular level

**Fig. 1:**
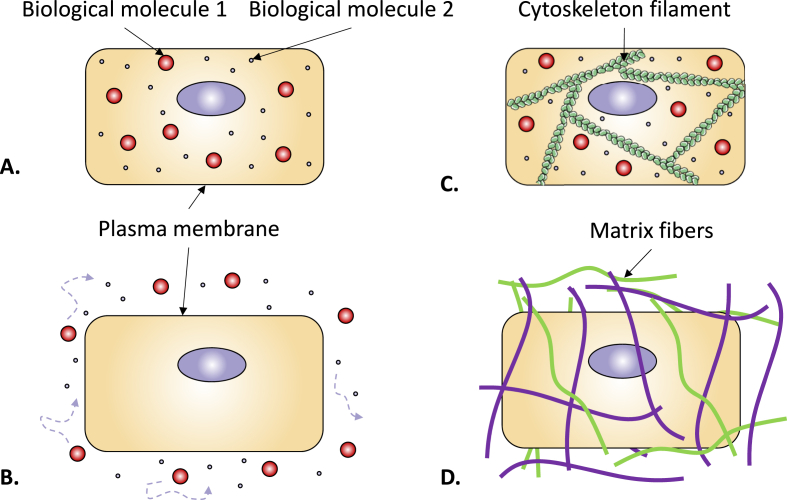
***Crowding and confinement in and out ofthecell.****High density of biological molecules in solution are found inside (A) and outside cells (B). High density of biological molecules in solution can lead to confinement due to specific molecules arrangement. Confinement arises from the presence of compartments located between rigid structures, such as intracellular actin filaments (C) and extracellular matrix fibers (D). Figure A and* C *are adapted from* Ref. [12].

The high density of macromolecules in biological milieu reduces the available space, giving rise to molecular crowding or even molecular confinement. Indeed, as a consequence of MMC, the arrangement of molecules leads to confinement [10], a process defined by the formation of microcompartments of objects in specific regions (Fig. 1). MMC arises from soluble macromolecules competing for space, while macromolecular confinement arises from fixed or semi-static structural barriers (e.g., cytoskeletal lattices, ECM fibers, lipid membranes) that restrict soluble molecule mobility [11]. For example, the cytoskeleton (Fig. 1C) and ECM fibers (Fig. 1D), can assemble into mesh-like frameworks whose pore size diminish as crowding increases, leading to molecular confinement due to an inability of the molecules to pass through the meshwork [10,12]. While both processes reduce the accessible space for macromolecules, their free energy landscapes differ. MMC promotes globally compact conformations (minimal radius of gyration) to minimize excluded-volume, while confinement fosters shape complementary to the geometry of the confining boundary. For instance, a spherical cavity stabilizes globular conformations, a cylindrical pore favors rod-like structures and a planar slit between two walls promotes plate-like morphologies [13,14]. Extensive reviews on the role of confinement are available [10,11,13,14].

**Fig. 2:**
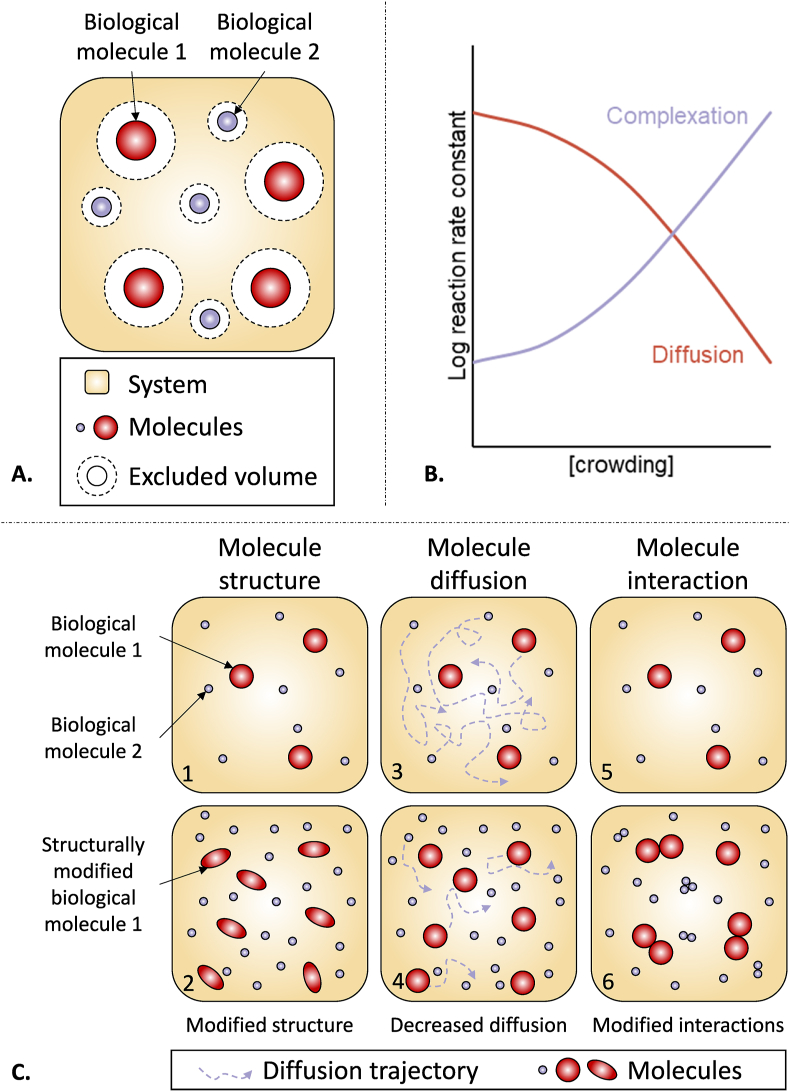
***Crowding at molecular level.****(A) The excluded volume effect is illustrated. A white area around each molecules show the excluded volume, therefore limiting mobility for any newly added molecules in the system. Other molecules cannot enter in the white area representing the excluded volume of each molecule. (B) Crowding decreases diffusion while increasing complexation of molecules. (C) Illustration of the consequences of crowding atthemolecular level. The lower left panel (2) shows that molecules conformation is affected by crowding compared tothediluted environment on the left upper panel (1). On the lower middle panel (4), molecules diffusion is decreased inthecrowded environment compared tothediluted environment on the upper middle pane (3). The right panels show that crowding differentially affect molecules interactions: compared to diluted media (5), in crowded media (6) two close molecules interaction is facilitated while interaction between two distant molecules from each other is decreased. High molecular weight molecules are more affected than smaller molecules. Adapted from* Refs. [12,17].

The presence of numerous macromolecules reduces the amount of free space in the environment, a phenomenon known as the excluded volume effect (Fig. 2A) [15]. This effect is largely discussed and results from a combination of steric repulsions and soft interactions [16]. In a homogeneous solution, molecules of equal size collide but maintain a minimum center-to-center distance of twice their radius. When a test molecule of similar size is introduced into this environment, it experiences equivalent steric interactions, thereby limiting its available space [10]. This leads to steric repulsion and a reduction in the translational degrees of freedom of solutes. MMC more significantly restricts the movement of solutes that are similar in size to, or larger than, the crowding agent, while having a lesser impact on the mobility of smaller solutes, which can move more freely. Consequently, biochemical reactions may be either enhanced or reduced, and even suppressed by excluded volume effects [10].

Beyond steric repulsion, crowding agents also exhibit soft interactions, which are non-specific chemical interactions between macromolecules and crowding agents in a crowded environment. These interactions include dispersion forces, electrostatic forces, and hydrogen bonding, which can be either attractive or repulsive depending on the specific properties of the molecules involved [16]. If the crowding agent and the molecule have the same charge, repulsion enhances the excluded volume effect, whereas opposite charges result in attraction, thereby reducing the excluded volume effect [16]. Moreover, polyethylene glycol (PEG) exhibits hydrophobic interactions, whereas Ficoll and dextran engage in hydrogen bonding, which influences their interactions with intrinsically disordered proteins [18]. Since intrinsically disordered proteins lack hydrophobic residues, they form more favorable interactions with Ficoll than with PEG. In a cell-free system, these soft interactions can destabilize the helical segment of thyroid and retinoid receptor activators, as well as the CREB-binding protein [18]. In a cell-based system, dextran sulfate (DxS) in cell culture media promotes ECM deposition *via* electrostatic interactions [19,20].

Due to restricted space, MMC leads to more rapid and frequent collisions between molecules. As a result, MMC decreases diffusion rate and increases complexation reaction rate and, it influences molecule's structure and interaction (Fig. 2B and C). MMC influences the rates and equilibria of molecular interactions, such as protein-protein and protein-nucleic acid interactions, leading to changes in binding affinities and complex formation. As MMC affects molecular motility, it can also potentially alter reaction kinetics, transport processes, molecular distribution, and the availability of molecules for cellular functions like gene expression [21]. Furthermore, MMC can modify the thermodynamic properties of biological macromolecules, influencing protein folding and conformational dynamics. Consequently, enzymatic activities and substrate availability may be affected, altering reaction rates, substrate binding, and product release [22].

As highlighted in the review by Zhou et al., the effects of crowding on enzyme activity cannot be easily generalized [11]. MMC affects enzyme activity differently depending on the enzyme, the crowding agent, its size, and its concentration. For example, bone-specific alkaline phosphatase, a biomarker used to diagnose and assess the severity of metabolic bone diseases, experiences reduced hydrolysis rates in the presence of dextran and Ficoll [23]. In contrast, DNase I, an enzyme implicated in various diseases, exhibits enhanced activity under PEG-induced crowding, demonstrating the opposing effects of crowding on different enzymes [[24], [25], [26]]. Thus, the influence of MMC on enzyme activity is highly complex and context-dependent. Several research reviews summarize the biophysical effects of crowding on protein structure and function but, given that numerous biological functions at the cellular and tissue levels may be affected by MMC, further research is needed to fully elucidate the role of MMC in modulating the activity of specific proteins and enzymes [11,27].

## 4 Intracellular macromolecular crowding

### 4.1 Macromolecular crowding in the cytoplasm

**Fig. 3:**
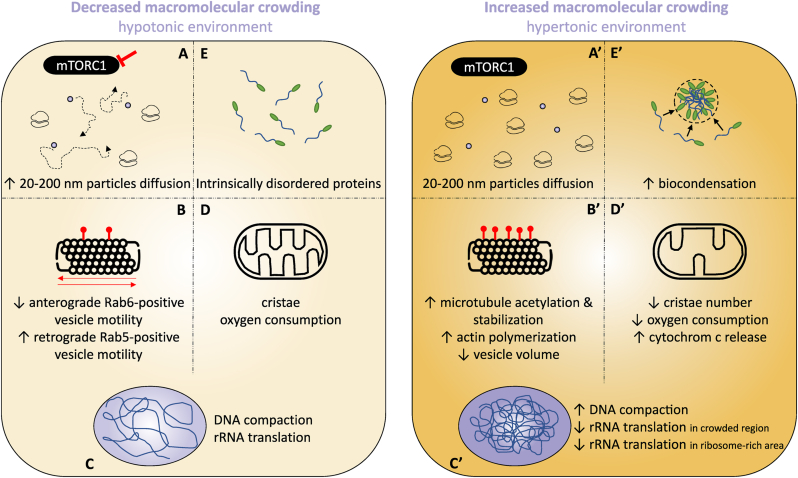
***Functional consequences of intracellular crowding.****Cytoplasmic lower crowding is correlated to lower ribosome concentration and thus increased 20–200* *nmparticles diffusion (A, A′)* [29]*. While lower crowding decreases anterograde vesicle motility and increases retrograde vesicle motility, higher crowding increases tubulin acetylation, microtubule/actin polymerization and decreases vesicle volume (B, B′)* [30,31,[32], [33]]*. Higher crowding in the nucleus increases DNA compaction and decreases rRNA translation in highly crowded areas, but rRNA translation increases in ribosome-rich regions (C, C′)* [34,[21], [35], [36]]*. Higher crowding in the mitochondria matrix decreases cristae number and thus decreases oxygen consumption while favoring cytochrom c release (D, D′)* [37,38]*. Higher crowding also promotes intrinsically disordered protein liquid-liquid phase separation to form biocondensate (E, E′)* [39,40].

Intracellular crowding significantly influences cellular processes, including trafficking and signaling pathways. The cytoplasmic protein concentration in human cells is estimated to be approximately 75 mg/mL [3]. Intracellular MMC increases in hypertonic microenvironments and decreases in hypotonic condition [28]. Delarue et al. demonstrates that mammalian target of rapamycin complex 1 (mTORC1), a cytoplasmic complex involved in the regulation of protein translation, modulates cytoplasmic crowding and gel-like properties by regulating ribosome concentration, thereby affecting particles diffusion [29]. Specifically, when mTORC1 activity is inhibited, ribosome concentration decreases, leading to an increase in the diffusion of particles ranging from 20 to 200 nm (Fig. 3A–A’).

Elevated intracellular crowding decreases vesicle and protein motility and disrupts cytoskeletal dynamics [30,41]. This effect may be linked to tubulin modifications, as increased intracellular crowding leads to higher levels of tubulin acetylation, resulting in microtubule stabilization (Fig. 3B–B’) [31]. Consequently, cytoplasmic crowding differentially regulates cargo transport: reduced crowding decreases the anterograde motility of Rab6A-positive secretory vesicles from the perinuclear region to the cell periphery while increasing the retrograde motility of Rab5-positive early endosomes [31]. Additionally, Sood et al. demonstrated that cargoes can become trapped in actin-rich regions of axons, generating local crowding and causing vesicles to stall at specific locations [42]. Axonal stimulation reduces the density of stationary presynaptic vesicles, suggesting that vesicles crowded in actin-rich regions serve as functional reservoirs that may contribute to maintaining a robust cargo flow in neurons [42].

Crowding influences actin filament formation by accelerating the polymerization of globular actin into filamentous actin [43]. Increased crowding correlates inversely with Young's modulus and filament length while positively correlating with bending stiffness [[44], [45], [46]]. Additionally, crowding reduces binding interactions between actin filaments and cross-linking proteins such as fascin and α-actinin [32]. Moreover, Ficoll-induced MMC increases the maximum velocity of the actomyosin ATPase cycle which is explained by a more compact conformation of the myosin head [47]. This model shows that crowding can have a significant impact in process like muscle contraction or cell trafficking and locomotion.

MMC also affects lipid membranes, leading to vesicles that are more compact than those in diluted environments, indicating a reduction in available volume within the vesicles [33]. MMC differentially affects vesicle-catalyzed production of decanoic acid *via* the hydrolysis of decanoic anhydride and vesicle-catalyzed production of oleic acid *via* the hydrolysis of oleic anhydride [48]. Crowding induced by PEG decreases the production rate of decanoic acid while increasing the production of oleic acid. These variations in production rates may be attributed to the increased size of decanoic acid vesicles and the reduced size of oleic acid vesicles. Given that the digestion and absorption of fatty acids in the intestine remain poorly understood, this model provides valuable insights into the autocatalyzed production of fatty acids.

### 4.2 Macromolecular crowding in the nucleus

The estimated average concentration of proteins in the HeLa nucleoplasm is approximately 150 mg/mL [4]. Increased intracellular crowding leads to reversible chromatin compaction and separation from the nuclear membrane affecting DNA accessibility to the transcriptional machinery which also influences gene expression, ribogenesis, and RNA synthesis (Fig. 3C–C’) [34,35].

Highly crowded nuclear compartments remain accessible to diffusing proteins and enhance the binding of chromatin-interacting proteins [49]. A study using a cell-free system demonstrates that 30 % crowding induces a reduction of at least 20 % in final mRNA production and leads to a complete loss of translational activity [21]. This crowding effect is associated with the emergence of distinct regions containing very high mRNA concentrations [21]. In this cell-free system, 30 % crowding was sufficient to completely inhibit translation. However, in cells, translation persists even at higher crowding fractions. This continued translation activity under high crowding conditions in cells is likely attributable to the heterogeneous nature of intracellular crowding. Specifically, translation may be minimal in highly crowded regions but remains active in less crowded or ribosome-rich areas of the cells [36]. Similarly, in a cell-free system, MMC enhances the activity of primase and helicase for DNA replication [50]. Additionally, crowding plays a crucial role in maintaining the nuclear localization of histone deacetylase, preventing its cytoplasmic leakage [51].

### 4.3 Macromolecular crowding in mitochondria

The total mitochondrial matrix protein concentration can be as high as 270–560 mg/mL [52]. Fluorescence recovery after photobleaching (FRAP) studies also reveal that the mitochondrial matrix is a crowded environment [37]. The diffusion of AcGFP1 (basic green fluorescent protein) in the mitochondrial matrix is approximately 23.9 μm^2^/s, which is about four times slower than in an aqueous solvent. Upon applying chloramphenicol to inhibit mitochondrial RNA translation, Bulthuis et al. observe an increase in mitochondrial matrix crowding, denoted by an increased viscosity, without any change in mitochondrial volume. Although the underlying mechanism of this increased crowding remains unclear, their study demonstrates that further intramitochondrial crowding reduces molecular diffusion even more by increasing matrix viscosity. Increased crowding in the mitochondrial matrix also reduces the number of cristae and decreases mitochondrial oxygen consumption, ultimately affecting overall mitochondrial function (Fig. 3D–D’) [37]. As several studies report a loss of cristae in skin fibroblasts from patients with mitochondrial DNA mutations, in Pseudoxanthoma elasticum dermal fibroblasts, and in peripheral blood mononuclear cells from patients affected by COVID-19, this study suggests that bioreactions in functional and dysfunctional mitochondria may be regulated by mitochondrial matrix crowding [[53], [54], [55]].

### 4.4 Macromolecular crowding and compartmentalization of biomolecular condensates

Biomolecular condensates (BCs) are membrane-less organelles formed through liquid-liquid phase separation (LLPS); a density transition similar to oil/water emulsions [56]. They are primarily composed of intrinsically disordered proteins. Environmental crowding modulates BC organization, density, compaction, and protein interactions [57].

The most well-known BC is the nucleolus, which initiates ribosome biogenesis through rRNA transcription and maturation in the nucleus [58]. The estimated average concentration of proteins in the nucleolus is approximately 284 mg/mL [4]. Crowding promotes the formation of larger condensates, as observed for the nucleolar protein Nucleophosmin (NPM1) and rRNA through microscopy analysis [39]. In a crowded cell-free environment, NPM1/rRNA biocondensates formation requires lower concentration of NPM1 and rRNA. Fluorescence resonance energy transfer (FRET) experiments show that increased crowding causes NMP1/rRNA condensates to reversibly transition from liquid to gel-like state as NMP1 and rRNA become immobile within the condensates. Similarly, crowding promotes phase separation of NPM1 with SURF6 (Surfeit locus protein 6) in a cell-free system and reduces diffusion within BC [40]. In cells, increased intracellular crowding also promotes the formation of RNA stress granules (Fig. 3E–E’) [59].

Thus, crowding can enhance LLPS, alter the biophysical properties of BCs, lower the critical protein concentration required for phase separation, and induce crowder co-condensation or partitioning into condensates [17]. Several comprehensive reviews summarize the effect of crowding on LLPS and biomolecular condensates [17,60].

## 5 Extracellular macromolecular crowding

### 5.1 Relevance of extracellular macromolecular crowding

**Fig. 4:**
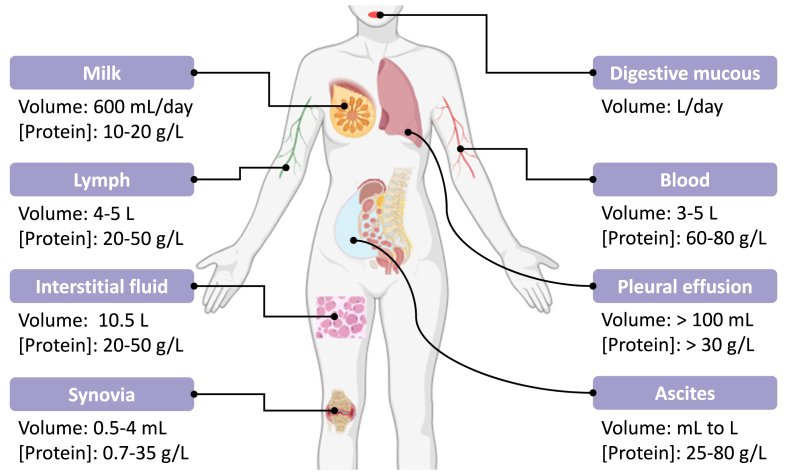
***Crowding in biological liquids.****Main biological liquids found in the human body and their respective protein concentration are represented* [2]*. Adapted from Biorender.*

Extracellular compartments, like their intracellular counterparts, are also densely packed with macromolecules (Fig. 4) [2]. Various physiological fluids in organisms have distinct compositions and macromolecular concentrations. Blood has the highest protein content, approximately 70 g/L, which is about ten times higher than that of typical culture media which contain only 0.5–10 % serum. Lymph and interstitial fluid contain between 20 and 50 g/L of proteins, while synovial fluid and milk can contain up to 30 g/L. In contrast, ocular fluid and cerebrospinal fluid have relatively low protein concentrations, with a maximum of 12 g/L and 3 g/L, respectively. The intestinal mucus layer is also highly crowded and forms a mucous membrane over the epithelial layer. Several liters of gastrointestinal mucus are secreted daily which consist of approximately 5 % mucin, 37 % lipids, 39 % proteins, and 6 % DNA by dry weight as determined by density-gradient centrifugation [61].

Pathological fluids can also accumulate under specific conditions. Ascites, which can build up in the abdominal cavity in cases of ovarian cancer, liver cancer, or cirrhosis, may contain up to 80 g/L of proteins [7]. Pleural exudative effusions, which occur in conditions such as pneumonia, cancer, tuberculosis, or pulmonary embolism, can contain more than 30 g/L of protein [62]. Edema fluid, formed due to increased vascular permeability, can contain up to 20 g/L of protein [63]. By mimicking the crowding conditions of these physiological and pathological fluids, relevant insights can be gained into cellular physiology and pathology within different tissue environments.

### 5.2 Extracellular macromolecular crowding and cell responses

Extracellular microenvironment plays a crucial role in ECM organization, biological reactions at the cell surface, and related cellular behaviors. Thus, extracellular MMC can significantly influence cellular responses. However, systematic studies on its effects on physiology and pathologies remain limited. Crowding enhances enzymatic reactions at the cell surface by reducing diffusion and increasing enzyme activities and binding rates [22]. It also increases local concentrations of growth factors and proteins, promoting autocrine signaling and ECM deposition [64,65]. Consequently, extracellular MMC accelerates ECM deposition [[66], [67], [68]]. In turn, increased ECM deposition also regulates cell fate as a biological molecule's reservoir [65]. MMC induces alignment of ECM fibers (fibronectin, collagen IV) and increases deposition of collagen I in mesenchymal stem cells. Concomitantly, an increased alignment of the cytoskeleton is observed. MMC also enhances mesenchymal stem cells proliferation while decreasing cells migration [69].

Current literature primarily exploits these properties of extracellular MMC in the context of tissue engineering. Still, to better understand the impact of extracellular MMC on cellular responses in physiopathological conditions, it is essential to develop body fluid-specific crowded culture media. This approach would enable more accurate studies of MMC effects on various cell types, including vascular, hepatic, and lymphatic cells (blood and lymph), interstitial cells (interstitial fluid), enterocytes (intestinal mucus), and cancer cells (ascites) among others. Effect of MMC on cell responses in physiological or pathological conditions is more extensively described in the following sections.

## 6 *In vitro* engineering of macromolecular crowding

### 6.1 Models to mimic macromolecular crowding and their specifications

As noted by Shadid et al., replicating the complex environment of biological fluids requires an ideal crowding agent that provides a natural microenvironment [70]. While using biological fluids such as ascites, blood, or cytoplasmic liquid would be optimal, their biological macromolecules heterogeneity complicates the interpretation of experimental data [71,72]. For instance, ovarian cancer cells (OV-90) exhibit heterogeneous responses to different ascites in terms of invasion, proliferation, spheroid formation, and gene expression [73]. To address the challenges posed by the heterogeneity of biological fluids, a "bottom-up" reconstruction approach is employed to mimic MMC induced by high-molecular-weight molecules in fluids and to understand its physical impact. This approach involves adding polymers to experimental setups to simulate MMC.

The most commonly used polymers for mimicking MMC are DxS, dextran (Dx), Ficoll, PEG and Polyvinylpyrrolidone (PVP). DxS is a branched polymer, generally 500 kDa, with a hydrodynamic radius of approximately 46 nm. It is negatively charged and is used at concentrations ranging from 1 to 100 μg/mL to facilitate ECM deposition through electrostatic interactions independent of MMC [19,74]. Dx is a linear charge-neutral polymer with few branches. Its hydrodynamic radius varies from 1.86 to 13.5 nm for molecular weights ranging from 9.5 to 500 kDa [75,76]. Ficoll is a branched, charge-neutral polymer. Since intracellular and extracellular biological fluids contain a variety of biomolecules with different hydrodynamic radii, Ficoll is often used as a binary mixture of two molecular weights: Ficoll 70 kDa and Ficoll 400 kDa, with hydrodynamic radii of 4.06 nm and 7.26 nm, respectively [77]. Ficoll is typically used at a blood physiological protein concentration around 62.5 mg/mL [69]. PEG and PVP are also commonly used to mimic MMC [39,[78], [79], [80]]. PVP 40 kDa and 360 kDa, as well as PEG 1–8 kDa, are linear charge-neutral polymers with hydrodynamic radii of 5.1 nm, 19 nm, and less than 3 nm, respectively [39,78,81]. All these polymers are relevant for MMC studies, as their hydrodynamic radii are similar to those of biological macromolecules found in blood, which range from 3.18 to 12.65 nm [82].

The size and shape of crowders also influence their effects on protein and DNA conformation as well as diffusion. Several reviews discuss the relationship between crowder properties and protein stability, structure, and activity [11,70]. Larger crowders generally stabilize proteins through volume exclusion effects, whereas smaller crowders can have more complex effects, sometimes destabilizing proteins through soft interactions, as observed for cytochrome *c* and N-terminal Src homology 3 [83,84]. Mardoum et al. demonstrated that branched and compact crowders, such as 10 kDa branched-PEG and 420 kDa Ficoll, cause DNA compaction, whereas linear and flexible crowders, such as 10 kDa and 500 kDa Dx, induce DNA elongation [85]. This size-dependent interplay between volume exclusion and soft interactions, along with shape-dependence, can be exploited to discern the effects of crowding on biological molecules under cellular conditions.

A study by Biswas et al. on human serum albumin showed that mixing different sizes of the same crowder (dextran 6–70 kDa) altered protein domain movements differently compared to a single-sized crowder solution or a mixture of different types of crowders (Dx and Ficoll, Dx and PEG): the separation between domains I and II increases with the increasing Dx 6 kDa concentration (∼75–100 g/L) and decreases with the increasing concentration of Dx 40 kDa, Dx 70 kDa or Ficoll 70 kDa; the interdomain separation undergoes an increase for the binary mixtures of Dx 6 kDa with either Dx 40 kDa, Dx 70 kDa, or Ficoll 70 kDa indicating that higher molecular weight Dx and Ficoll effect override the effect of low molecular weight Dx 6 kDa [86]. These findings indicate that further studies are needed to determine the optimal combination of crowding agents for structural and cellular research.

In addition to synthetic polymers, several natural polymers are also used, including alginate, carrageenan, fucoidan, and albumin [87,88]. In cell-free systems, albumin has been shown to reduce the aggregation and fibrillation of proteins, including alcohol dehydrogenase, insulin, transthyretin, and islet amyloid polypeptide [89,90]. However, adding these crowders to culture media to mimic extracellular MMC can complicate data interpretation due to their inherent biological activity, which may interfere with the physical crowding effect. Hyaluronic acid has also been tested but was found to be less effective than Ficoll or carrageenan, particularly in promoting ECM deposition [91].

Most studies investigating MMC use cell-free systems to examine protein and nucleic acid structure, interaction, and function under crowded conditions. Some studies also culture cells in polymer-crowded environments to emulate extracellular liquid crowding. As described in later sections, extracellular MMC is primarily applied in tissue engineering and the maintenance of sensitive cells. However, only a limited number of studies use extracellular MMC to model tissue physiology and pathologies, highlighting the need for further research in this area.

**Fig. 5:**
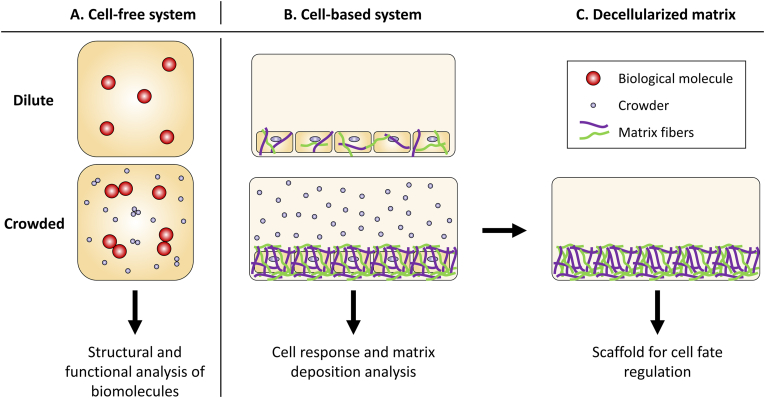
***In vitro models ofcrowding.****On the left, a cell-free system is represented (A). The upper panel shows a dilute solution of a biological molecule (brown). The lower panel represents the same solution of a biological molecule (brown) in presence of smaller molecule (purple). The cell-free models of crowding are generally used for structural and functional studies of protein, nucleic acid and lipids (not exclusively). On the middle panel, a cell-based system is represented (B). The upper panel represents cells cultured in a dilute media with extracellular matrix deposition (green and purple filaments). The lower panel represents cells cultured in presence of crowders (purple circles) and the increased extracellular matrix deposition (green and purple filaments). The matrix produced under crowded environment can be decellularized and used as scaffold for other cell types culture (C). The cell-based models of crowding are generally used for cell behaviors analysis and extracellular matrix analysis. (For interpretation of the references to colour in this figure legend, the reader is referred to the Web version of this article.)*

To summarize, DxS, Dx, Ficoll, PEG, and PVP are all biocompatible polymers. DxS is the only negatively charged polymer among them, while Dx, Ficoll, PEG, and PVP are electrically neutral, making them ideal for studying crowding effects without charge-based interactions. Additionally, Ficoll and Dx have highly branched, spherical structures that more closely resemble the globular proteins found in cellular environments compared to the linear structures of PEG and PVP. The choice of crowding agent depends on the specific research goals, the properties of the agent, and its compatibility with the experimental system. For structural studies, the neutral and well-characterized effects of PEG and Dx are preferred. In contrast, Ficoll, DxS, and PVP are more commonly used in cell culture applications, with Ficoll being particularly favored due to its globular structure and neutral charge compared to polyanionic DxS (Fig. 5). However, this distinction is not absolute, and further research, along with careful consideration of the specific properties of crowding agents, is needed to enhance our understanding of MMC and its impact on cellular processes.

### 6.2 Extracellular macromolecular crowding for tissue engineering and cell maintenance

Extracellular MMC can be used for tissue engineering either to accelerate ECM deposition by cells or to produce tunable gels for cell culture. Indeed, collagen hydrogels can be assembled in the presence of increasing concentrations of Ficoll 400 kDa and exhibit thinner collagen fibril diameters and decreased pore sizes [92]. These gels are more resistant to collagenase and demonstrate higher mechanical resistance to indentation. Furthermore, they are more favorable for mesenchymal stem cell proliferation on top of the gel. In another study, tunable collagen matrices formed in presence of PEG showcase physical properties similar to those produced with Ficoll: decreased pore size, shorter collagen fiber length, and lower degradation by matrix metalloproteinase-8 (MMP-8) [80]. MDA-MB-231 breast cancer cells encapsulated within the gels exhibit collective morphogenesis behavior rather than single-cell behavior compared to gels formed in a dilute environment. These studies demonstrate that MMC can be used to enhance polymerization and fine-tune hydrogel properties in cell-free systems.

Extracellular MMC accelerates ECM deposition by various cell types, including fibroblasts and mesenchymal stem cells, enabling the creation of dense, functional tissue substitutes. This effect is of significant interest for tissue engineering as it can accelerate *in vitro* patient autologous tissue production, which typically requires 196 days for tissue and functional repair [68]. Utilizing MMC to facilitate ECM deposition creates a dynamic cell-matrix reciprocity that, in turn, influences cellular phenotype and function—an essential characteristic for tissue engineering [65]. Robust cell sheets of 3T3 fibroblasts, human bone marrow mesenchymal stem cells, and hepatic cell sheets on top of 3T3 fibroblast layers can thus be generated under crowded conditions [93]. ECM produced under crowded conditions can be decellularized and used as a scaffold and signaling reservoir to modulate the fate of cells seeded on the decellularized matrices (Fig. 5).

MMC facilitates ECM deposition, and particularly collagen I, in cell culture, by several cell types [9,94]. The enhanced deposition of collagens (I, III, IV, V, VI) matrix by corneal fibroblasts enables the production of dense and cohesive living substitutes within six days, with tissue-specific functions ideal for tissue engineering [95]. Extracellular MMC is also a candidate for tendon tissue engineering as carrageenan crowder promotes collagens (I, III, IV, V, VI) deposition and promotes tendon markers expression (scleraxis and tenomodulin) by human tenocytes [96,97]. Several publications on scar-in-a-jar models replicate fibrosis-like scar tissue *in vitro* using MMC [[98], [99], [100], [101]]. These models demonstrate increased collagen deposition and cell fibrosis markers expression and, are utilized for drug efficacy screening, as described in the section "Macromolecular Crowding in Drug Delivery and Screening."

Beyond collagen I, MMC promotes the deposition of other proteins, including fibronectin by fibroblasts, fibrillin-1 by lung fibroblasts and Marfan syndrome patient-derived fibroblast cell lines, and basement membrane proteins such as collagen IV and laminin 1 by gingival fibroblasts [[102], [103], [104], [105]]. Notably, Satz-Jacobowitz et al. achieved faster and enhanced fibrillin-1 deposition in the form of elastic microfibrils which is a challenging task working only with hyperconfluent cells [103]. Mutations in collagen and fibrillin-1 can lead to deposition defects in osteogenesis imperfecta and Marfan syndrome [103,[106], [107], [108]]. Satz-Jacobowitz et al. thus emulate physiological conditions in normal and Marfan syndrome patient-derived fibroblast cell-lines cultures to investigate differences in fibrillin-1 deposition patterns. The ability of MMC to enhance fibrillin-1 deposition in some Marfan syndrome patient-derived fibroblast cell lines, compared to the abnormally low deposition in dilute media, suggests potential applications in tissue engineering for tendon or vascular tissue generation [103].

Moreover, MMC enhances the production of enzymes that cross-link collagen, such as lysyl oxidase and transglutaminase, contributing to ECM stabilization [79,94,109]. However, MMP activity may also be stimulated, leading to remodeling of the deposited ECM [97,110]. Thus, MMC boosts supramolecular assembly of ECM proteins but is also a regulator of ECM deposition and remodeling by modulating enzymatic processing.

MMC can also facilitate the long-term culture of difficult-to-maintain cells, such as stem cells, podocytes, and neuronal cells. MMC maintains the multilineage potential of mesenchymal stem cells while also modulating their differentiation responses upon specific stimulation [68,110,111]. When cultured under MMC with Ficoll, bone marrow mesenchymal stromal cells exhibit enhanced adipogenic or chondrogenic differentiation under respective adipogenic and chondrogenic stimulation [111].

As mentioned earlier, MMC can be used to enhance ECM deposition, and the ECM produced under MMC conditions can be decellularized to serve as matrix scaffolds for modulating cell-ECM interactions. Based on this principle, ECM produced under MMC by fibroblasts and mesenchymal stem cells, and subsequently decellularized, supports the maintenance of pluripotency in human embryonic stem cells and the expansion of hematopoietic stem/progenitor cells [109,112]. Similarly, ECM produced by ARPE-19 retinal pigment epithelial cells under MMC conditions can also be decellularized and used to promote retinal pigment epithelium phenotype and pluripotent stem cell differentiation [64]. The study of Benny et al. uses MMC to accelerate skin culture development *in vitro* [105]. Ficoll increases ECM deposition (collagen I, IV, VII) by dermal fibroblasts. ECM of dermal fibroblasts are then decellularized and, keratinocytes seeded on top of them could produce collagen VII while they could not when cultured as monoculture with MMC. When keratinocytes are seeded on collagen gel containing dermal fibroblast and supplemented with Ficoll, the skin substitute expressed collagen VII faster than the equivalent cultured without Ficoll.

MMC can also be used to aid the culture of podocytes, highly specialized kidney cells responsible for blood filtration [113]. ECM produced under MMC conditions by fibroblasts for ten days can be decellularized and used as a scaffold for podocyte culture. Podocytes cultured on MMC-produced matrix scaffolds display increased expression of molecules such as synaptopodin and nephrin, which are crucial for their structure and function, and develop intercellular connections, consistent with their physiological maturation [113].

An even more promising application of MMC is in neuronal cell culture, which is notoriously difficult [114]. MMC induced by Ficoll enhances the deposition of relevant brain ECM (collagen IV, fibronectin, and laminin) by cultured human induced pluripotent stem cells (iPSC)-derived astrocytes (iAstro). Human iPSC-derived dopaminergic neurons (iDopa) are then seeded on top of the iAstro-ECM bed and co-cultured for two weeks. The iDopa cells thus exhibit several notable improvements: longer neuronal extensions, increased synaptic vesicle numbers, an increased number of pre- and post-synaptic contacts, and enhanced calcium dynamics. These findings highlight the significant impact of MMC on both astrocyte ECM production and subsequent neuronal function, leading to neural cultures that more closely mimic physiological conditions [114].

## 7 Macromolecular crowding in health and disease

The beneficial effects of extracellular MMC in tissue engineering are currently being extensively studied as it can accelerate *in vitro* autologous tissue production and improve sensitive cells culture. Several publications demonstrate the impact of crowding on pathological protein polymerization in cell-free systems. However, limited research is conducted on harnessing MMC influence on cells under physiological conditions or diseased cells in pathological conditions. This section focuses on the few available studies examining the effects of extracellular MMC on cells in both physiological and pathological contexts. We first discuss how biological liquid crowding may be altered in specific diseases, followed by an exploration of MMC's impact on pathological protein polymerization, disease-associated bacterial behavior, osteoarthritis, cancer, and vascular physiopathology. In the final part, the use of MMC in drug delivery system development and its application in creating fibrosis-like tissue *in vitro* for anti-fibrotic drug screening are highlighted.

### 7.1 Modification of macromolecular crowding in health and diseases

*In vivo*, cells are constantly exposed to highly crowded liquid environments, unlike the diluted media typically used *in vitro*, which contain only 0.5 %–10 % serum, corresponding to 0.4–8 mg/mL of proteins. This discrepancy introduces a bias in cell culture experiments. Most cells are in continuous contact with interstitial fluid. Specialized cells, such as vascular endothelial cells and mammary epithelial cells, interact with highly crowded fluids such as blood and milk (Fig. 4) [2]. Pathologies such as ovarian cancer, stomach cancer, and cirrhosis are associated with abnormal accumulation of ascitic fluid, which has high extracellular MMC in the abdominal cavity [115]. As a result, both cancerous and healthy cells in the abdominal cavity interact with highly crowded liquid environments.

Hyperproteinemia, observed in conditions such as multiple myeloma, HIV infection, and chronic kidney disease, leads to elevated protein concentrations [116]. Conversely, hypoproteinemia, associated with malnutrition or acute respiratory distress syndrome, results in decreased protein levels [117,118]. In osteoarthritis, synovial fluid has been shown to exhibit lower osmolarity and higher protein concentrations due to inflammation, which increases intracellular crowding and alters signaling pathways [[119], [120], [121]]. Additionally, pathologies associated with altered protein polymerization, such as fibrosis and neurodegenerative disorders, are characterized by increased expression, production, or accumulation of specific proteins, leading to pathological polymerization [122]. These variations in protein concentration result in modifications of both extracellular and intracellular crowding, potentially affecting cell fate.

### 7.2 Macromolecular crowding and pathological protein polymerization

Protein misfolding, aggregation, and accumulation are hallmarks of protein misfolding diseases and are associated with various pathologies, including fibrosis, type II diabetes mellitus (T2DM), prion, Alzheimer's, Parkinson's, and Huntington's disease. A crowded environment plays a crucial role in protein aggregation *in vivo*. Proteins are more likely to misfold and aggregate under crowded conditions due to altered folding pathways and increased effective concentrations, leading to the formation of toxic aggregates such as amyloid fibrils and, potentially, cell death [123].

Crowding promotes virus-like particle assembly, suggesting that intracellular MMC may facilitate viral amplification in host cells [124]. *In vitro* cell-free studies have shown that crowding accelerates amyloid formation and aggregation of various proteins, including apolipoprotein C-II, which is implicated in amyloidosis, and hemoglobin [125,126].

Several studies demonstrate that MMC promotes ECM deposition, particularly collagen I, which is associated with fibrosis markers [[98], [99], [100], [101]]. Collagen deposition is accelerated by myofibroblasts under PVP 40 kDa-induced MMC and by human fibroblasts under Ficoll- and Dx-induced MMC [[98], [99], [100], [101]]. A cell-free model confirmed that Dx and Ficoll enhance collagen fibrillization in a concentration-dependent manner [127].

Islet amyloid polypeptide (IAPP), a protein involved in hyperglycemia regulation, is co-secreted with insulin. In T2DM, the loss of insulin control coincides with the loss of β-cell mass and the deposition of IAPP aggregates [128]. The misfolding of IAPP is implicated in the degeneration of pancreatic islets [128]. Crowding agents such as Dx, Ficoll, and PVP differentially affect IAPP aggregation. PVP 360 kDa promotes fibril formation due to excluded volume effects, while PVP 10 kDa inhibits fibril elongation by reducing elongation rates [129]. Increased crowding by Ficoll 70 kDa, Dx 70 kDa, BSA, and lysozyme also reduces IAPP fibril formation [90]. Correlated with this reduction, cytotoxicity of IAPP on the pancreatic INS-1E β-cell line was also low in a crowded environment.

In Parkinson's disease, α-synuclein polymerizes into amyloid aggregates/fibrils within neurons, which are released into the extracellular compartment, ultimately leading to neuronal death [130]. Both primary nucleation and fiber elongation of α-synuclein amyloid formation are accelerated in a Ficoll 70 kDa-crowded environment [131]. This fibrillization is also enhanced by PEG, Dx, lysozyme, and albumin, with higher molecular weight PEG further accelerating α-synuclein polymerization [132].

Alzheimer's disease is characterized by the accumulation of amyloid β (Aβ) and Tau proteins, which form insoluble fibrils [133]. Dx 70 kDa and Ficoll 70 kDa increase the rate of Aβ nucleation in the presence of agitation [134]. Similarly, Ficoll 70 kDa and Dx 70 kDa promote the fibrillation of Tau fragments, with Dx exerting a stronger effect than Ficoll [[135], [136], [137]].

Huntington's disease is a fatal neurodegenerative disorder caused by an expansion of the polyglutamine domain in the first exon of the huntingtin (Htt) protein, which is directly implicated in toxic aggregate formation [138]. Crowding induced by Dx 20 kDa and Ficoll 400 kDa increases Htt fibril formation in solution [139]. Interestingly, crowding notably decreases Htt aggregation on mica surfaces, indicating an influence of crowding on Htt-interface interactions [139].

The misfolded and aggregated state of prion protein (PrP) is associated with prion diseases, including Creutzfeldt-Jakob disease [140]. Increased crowding by Ficoll 70 or 400 kDa is shown to enhance amyloid fibril formation of both wild-type human PrP and its pathogenic mutants E196K and D178N, with the effect being stronger on the mutants [136,137]. Additionally, crowding induced by Ficoll 70 kDa and Dx 70 kDa enhances fibril formation of human and bovine PrP, both being susceptible to prion disease, while it inhibites fibril formation of rabbit PrP, which is resistant to prion disease [141]. This inhibition of fibril formation correlates with the higher α-helix and lower β-sheet content in rabbit PrP compared to human and bovine PrP. Moreover, Ficoll 70 kDa-induced crowding promotes the formation of short, protease-resistant human PrP fibrils, unlike those formed in dilute solutions [136].

In summary, crowded physiological environments might play a crucial role in cellular quality control mechanisms by regulating protein folding and aggregation both intracellularly and extracellularly. The effects and mechanisms of MMC on protein conformation, aggregation, and activity are summarized in several review articles [123,142,143].

### 7.3 Macromolecular crowding and disease-associated bacteria and biofilms

Microbiota refers to the collection of microorganisms that reside on and within the human body. Disruptions in microbiota equilibrium are associated with various pathologies, including uropathies, inflammatory bowel diseases, and cystic fibrosis. The respiratory and gut microbiota are continuously in contact with mucus layers [[144], [145], [146], [147], [148]]. The mucus layer is a complex and dense structure composed of various macromolecules, including mucins, proteins, lipids, and other biomolecules. This composition creates a physically crowded microenvironment that influences bacterial behavior and host-microbiota interactions [149]. Similarly, bacteria can self-organize into multicellular communities known as biofilms, in which cells are embedded in a self-secreted extracellular polymeric matrix composed of polysaccharides, nucleic acids, and proteins [150]. This structure also creates a crowded environment that impacts bacterial survival and behavior.

Bacterial cells, such as *Escherichia coli*, exhibit intracellular MMC, occupying 15 %–20 % of the cell volume, which is modulated by environmental conditions. Antibiotic treatments, such as penicillin, increase intracellular MMC by disrupting bacterial cell walls, leading to nucleoid dispersion within bacteria [151]. This study suggests that manipulating intracellular crowding in bacteria could be a potential strategy to disrupt bacterial infections or biofilm formation.

Extracellular mucus in the gut or respiratory also impacts microbiota [145,147,149]. A review summarized the biophysical forces influencing gut biofilm formation, including the impact of extracellular molecules in the ECM: (i) extracellular polymers maintain a distance from the cell surface shorter than their radius of gyration around aggregated bacteria, (ii) extracellular polymers link bacteria together *via* electrostatic interactions, and (iii) the crowded mucus layer may exert compressive forces on bacteria, potentially upregulating extracellular polymer production by uropathogenic *Escherichia coli* [145].

Alginate-induced crowding (a major exopolysaccharide associated with the mucoid phenotype of *Pseudomonas aeruginosa* strains isolated from cystic fibrosis patients) increases the relative growth rate of FapC amyloid assembly and leads to morphological polymorphic FapC fibrils important in cell adhesion. This highlights the importance of studying bacterial and biofilm formation in a more physiologically relevant crowded environment [152]. Computational models show that self-produced extracellular polymeric matrices induce crowding and repulsive interactions, leading to spontaneous bacterial aggregation [149]. Another computational simulation demonstrates that crowding conditions in biofilms reduce effective diffusion, enhance metabolite concentration, and affect nutrient supply, waste diffusion, and chemical treatment efficacy [153]. Within biofilms, crowding promotes the development of nutrient concentration gradients, allowing bacteria at the biofilm periphery superior access to nutrients, while those in the core experience increased interaction with waste products [153]. Given that biofilms are known to promote antibiotics resistance and are inherently crowded environments, the impact of crowding on antibiotic diffusion and efficacy warrants further investigation [154].

Increased viscosity from crowding agents like Ficoll enables bacteria such as *Shewanella putrefaciens* to escape entrapment using unique flagella structures known as screw-like flagella [155]. This bacterium wraps its flagella around its cellular body and, by rotating them, can escape from entrapment. Higher Ficoll content increases the fraction of bacteria exhibiting screw-like motion [155]. This study underscores how bacteria can alter their behavior in crowded environments, which may contribute to infection progression and antibiotic resistance.

Overall, MMC, both intracellular and extracellular, affects bacterial processes, community behavior, and microbial interactions with the host. Several diseases, including inflammatory bowel diseases and cystic fibrosis, are associated with bacterial infections. However, studies on the role of MMC in bacterial growth under these physiological and pathological conditions remain scarce. Further research is needed to develop more complex *in vitro* bacterial models to elucidate the dynamics of planktonic bacteria and biofilms and, to develop effective strategies for combating biofilm-associated infections.

### 7.4 Macromolecular crowding in osteoarthritis

Osteoarthritis is a degenerative joint disease characterized by reduced synovial fluid osmolarity, with osteoarthritic joints exhibiting approximately 300 mOsm compared to 400 mOsm in healthy joints [119]. This decreased osmolarity induces a reduction in intracellular MMC. Research by Govindaraj et al. demonstrates that chondrocytes, whether from healthy or osteoarthritic donors, experience higher intracellular crowding when exposed to healthy-like osmolarity [121]. Increased intracellular crowding enhances chondrocyte responses to pro-regenerative anabolic BMP7 and TGFβ signaling while diminishing their response to pro-inflammatory catabolic NFκB signaling, a pathway directly linked to inflammation [121]. Additionally, increased intracellular crowding promotes glycolysis as the primary energy metabolism pathway. These findings suggest that intracellular crowding plays a crucial role in stabilizing or destabilizing cellular anabolic, catabolic, and metabolic behavior, offering potential therapeutic strategies for renormalizing osteoarthritis-affected chondrocytes [121]. Osteoarthritis is also characterized by an increased protein concentration in synovial fluid, associated with a rise in inflammatory proteins [120]. However, the impact of increased extracellular MMC remains unexplored and requires further study.

Another study examined the effects of increased intracellular crowding under higher osmolarity in NIH-3T3 fibroblasts and HeLa cells. The results showed that increased intracellular crowding, compared to normosmolarity, leads to the activation of TNFR1 (tumor necrosis factor receptor 1) and NFκB [156]. Both the studies by Govindaraj et al. and Biswas et al. demonstrate that deviations from healthy intracellular crowding levels—either an increase or a decrease—trigger NFκB signaling. These findings underscore the importance of studying intracellular crowding alterations in various pathologies, as crowding modifications can activate NFκB signaling in healthy and osteoarthritic chondrocytes, HeLa cancer cells, and healthy NIH-3T3 cells.

### 7.5 Macromolecular crowding as a tool in cancer studies

Tumor progression involves cell dissemination through the vascular network and/or the accumulation of ascites in abdominal cavity. Little information is available about the direct relationship between extracellular MMC and unhealthy cell responses in tumor development.

In several cancer, ascites fluid accumulates within the abdominal cavity as a result of vascular and lymphatic leakage and compromise therapies efficacy [157]. MMC of ascites can increase oncotic pressure and ascites can affect cancer cell behavior [73,158]. For instance, liver cancer cells and cancer-derived endothelial cells respond to high-macromolecular-content solutions (alginate, Dx, PEG or PVP) with morphological changes and increased ECM deposition [159]. In contrast to normal human umbilical vein endothelial cells, the endothelial cell line SK-HEP-1 derived from hepatic adenocarcinoma elongated and aligned in a manner that was dependent of the molecular weight of the crowders. In addition, these morphological alterations were accompanied by an increase in ECM deposition [159].

Fibroids, tumors of the uterine myometrium composed of smooth muscle cells and ECM, can be modeled *in vitro* using MMC [160]. In a Ficoll-induced crowded environment supplemented with ascorbic acid, hTERT-HM myometrial smooth muscle cells increase collagen production, facilitate proteolytic removal of the C-propeptide from secreted procollagen and increase the secretion of fibrosis marker activin A [160].

MMC affects ovarian cancer cell adhesion, migration, and cytoskeletal organization, influencing cancer cells progression and dissemination. Ficoll and Dx induced-MMC decreases IGROV1 and SKOV3 ovarian cancer cell adhesion and spheroid formation in suspension, which might favor cell death by anoikis [7]. Meanwhile, MMC increases adherent ovarian cancer cell migration by decreasing ECM deposition, which might contribute to the progression and dissemination of adherent cancer cells [7].

As described in the section “Extracellular macromolecular crowding for tissue engineering and stem cell maintenance”, increased production of ECM under MMC creates a bidirectional cell-matrix dynamic which can regulate cell fate [65]. ECM produced under MMC and then decellularized can thus be used as a scaffold for cancer cells and be used as a tool to study tumor development on these matrices. Mammary fibroblast-derived ECM are thus produced under MMC and decellularized with detergent NP-40 [161]. Adenocarcinoma mammary gland MDA-MB-231 cells seeded on these matrices present significant increase in expression of focal adhesion molecules, matrix metalloproteinases and proinflammatory cytokines [161]. Furthermore, MDA-MB-231 cells seeded on these MMC-derived ECM significantly increase doxorubicin resistance and reduce the impact of intracellular reactive oxygen species-mediated cell death [161]. Further investigation is needed to understand the roles of MMC in tumor biology and epithelial-mesenchymal transition, to develop therapeutic strategies to manage tumor resistance and pathological ascites accumulation and, to improve patient outcomes.

### 7.6 Roles of macromolecular crowding in vascular physiology and pathologies

The crowded blood within vessels might significantly influence vascular function, cell behavior and contribute to various vascular diseases. Pathologies associated with hypo- or hyperproteinemia, as well as conditions like atherosclerosis, which involves increased cholesterol and triglyceride concentrations, can modify blood crowding. However, research on the effects of crowding on vascular biology is limited.

#### 7.6.1 Effects on blood cells

Red blood cells (RBCs), platelets (PLT), leucocytes and monocytes are constantly immersed in crowded blood. In the blood vessel, it is generally accepted that flow but also crowding induced by the presence of RBC concentrates PLT near the vascular wall rather than to the center of the vessel tube, confirming a role of crowding in the vascular system [162]. Ficoll-induced MMC decreases RBC sedimentation time, with concentration-dependent effects on RBC aggregation. At 5 % Ficoll 70 kDa crowding, RBC rouleaux formation is promoted, while at 10 %, this formation does not happen [163]. RBC aggregation is dependent on the crowder size, with 200–500 kDa Dx showing optimal aggregation effects at concentrations below 2 g/dL, mimicking hypoproteinemia [164]. Further investigation is needed to determine the effects of crowding on RBC in mixed crowder solutions that more accurately represent blood composition as blood is not composed of single size molecules.

PLT are major cells participating in coagulation and mechanosensing substrate stiffness with α_IIb_β_3_ integrins activation [165]. They are thus able to sense biochemical and physical properties but no studies have been performed on the role of MMC on PLT. Still, PLT study might be difficult as they are easily activated by plenty of stimuli (changes in oxygen, temperature, friction etc.) and form PLT aggregates. Further studies also need to be carried out on leucocytes and monocytes to determine the effect of MMC on their transmigration and activation during inflammation [166,167].

#### 7.6.2 Effects on vascular wall cells

Arterial walls are composed of three layers: (i) intima, composed of endothelial cells in contact with the blood, (ii) media, mainly composed of vascular smooth muscle cells (VSMC) in interstitial fluid, and (iii) adventice, constituted of fibroblasts in interstitial fluid.

In three-dimensional perfusable microfluidic devices, Ficoll-induced MMC stabilizes and improves the functionality of microvascular networks formed by umbilical endothelial HUVEC cells in 3D matrix [168]. MMC decreases the loss of cellular junctions between endothelial cells over time, enhances basal membrane laminin and collagen IV expression and deposition, increases adherent junction β-catenin and VE-cadherin expression, and reduces permeability to small molecules [168].

VSMC, responsible for vessel contraction, are also affected by MMC. VSMC linger in interstitial liquid. Dyslipidemia, characterized by an increase of blood cholesterol and triglycerides, is associated with endothelial dysfunction, allowing an increased leakage of blood components in the media [169]. In a model mimicking blood MMC using carrageenan, MMC demonstrated a dual role on VSMCs. While decreasing VSMC proliferation and migration, MMC maintained or increased contractile function of VSMC, with enhanced expression of contractile markers α-smooth muscle actin, calponin and transgelin [170].

Vascular pathologies like atherosclerosis are associated to VSMC switching toward other phenotypes including transition to macrophage-like, foam, or osteoblast-like phenotypes [169]. The role of MMC in VSMC phenotypic and functional switches during atherogenesis remains unexplored. Further research is needed to elucidate the impact of MMC supplemented with inducing factors on these phenotypic and functional switches, lipid deposition and immune cell infiltration in atherosclerotic plaques.

### 7.7 Macromolecular crowding in drug delivery and screening

MMC presents significant challenges for drug efficacy *in vivo*. The crowded environment can hinder drug diffusion, accessibility to target molecules, function of drug transporters for drug uptake and efflux, potentially limiting the effectiveness of treatments and contributing to treatment resistance.

The diffusion rates of small active compounds are influenced by self-aggregation, interactions with crowders, and surface adsorption [171]. Doxorubicin and SB216763 (a glycogen synthase kinase-3 inhibitor) diffusion rate are increased in presence of protein crowders like albumin and myoglobin by reducing respectively surface interactions and self-aggregation. In presence of the same crowders, quinacrine diffusion rate remains unaffected. In an *in vitro* reconstructed model of mucus with mucins, lipids and albumin, Lahred et al. also shows that hydrophilic mannitol diffusion remained unchanged, while lipophilic drugs metoprolol and propranolol (which carry a net positive charge at physiological pH) and testosterone and hydrocortisone (which are uncharged) diffusion is reduced (this reduced diffusion being attributed mainly to lipid and albumin) [61].

MMC can be used to develop and fine-tune drug delivery systems. MMC generated by PEG is shown to create stronger gels by gelator 9-fluorenylmethyloxycarbonyl-diphenylalanine [172]. The gels generated under crowded environment facilitate the controlled release of poorly water-soluble anti-cancer drugs such as Paclitaxel [172]. The drugs exhibit a gradual release profile from the hydrogel, subsequently diffusing into the aqueous medium driven by a concentration gradient, ultimately resulting in complete liberation of the drug molecules from the gel.

Reproducing pathological tissues for drug screening is crucial. MMC is used to mimic fibrotic-like and hypertrophic scar matrices *in vitro* called the scar-in-a-jar models. The scar-in-a-jar model more closely recapitulates *in vivo* conditions of increased collagen deposition, and allow the screening of anti-fibrotic agent [99,101]. Fan et al., thus demonstrates that shikonin and naphthazarin possess similar biological activity within a similar dose range increasing hypertrophic scar-derived human fibroblasts mortality in Ficoll-induced crowded environment [101]. MMC induced by DxS combined with TGFβ1 increases collagen deposition and α-SMA expression in fibroblasts, effectively recapitulating fibrosis-like conditions *in vitro* [99]. The recombinant protein T22d35, an inhibitor of TGFβ signaling, has no effect on collagen deposition, whereas T122bt, another TGFβ signaling inhibitor, significantly reduces collagen deposition. Triamcinolone acetonide, a histone deacetylase inhibitor, has only a moderate effect on collagen deposition in a crowded environment. This finding supports clinical data indicating that, despite its widespread use for treating hypertrophic scars, it has a moderate response rate [99]. Two other anti-fibrotic compounds significantly decrease collagen deposition in a crowded environment: the histone deacetylase inhibitor Trichostatin A and the pleiotropic inhibitor of fibrotic activation Serelaxin [99]. The discrepancy in the effects of anti-fibrotic compounds may be explained by MMC altering protein and receptor conformation, activity, and binding affinity [27]. Indeed, *in vitro* inhibition assays of PP1 (c-Src kinase inhibitor) conducted in a cell-free system (composed of c-Src kinase, Src, and ATP), under both dilute and albumin-crowded conditions, demonstrate that crowding reduces the inhibitory effect of PP1 on c-Src kinase by inducing a conformational shift of Tyr82 at the hinge region of c-Src kinase [173].

The scar-in-a-jar model was adopted by GlaxoSmithKline, and two of their publications indicate that MMC can be used to model idiopathic pulmonary fibrosis for potential industrial applications and anti-fibrotic drug screening [174,175]. The prolonged scar-in-a-jar assay, combined with clinically validated biochemical markers of ECM synthesis, enables the evaluation of ECM synthesis (collagen I, III, VI, fibronectin, α) over time and validates the model as a tool for screening novel anti-fibrotic compounds (nintedanib, pirfenidone, omipalisib). The adoption of the scar-in-a-jar model by GlaxoSmithKline thus represents a success story in the early industrial adoption of MMC for fibrosis drug screening.

MMC can be utilized for drug delivery and screening systems but further studies are required to determine whether crowding also impacts the delivery and efficacy of other drugs, such as anti-cancer and anti-coagulant drugs. Additionally, to fully understand drug efficacy in a crowded environment, further research is needed to assess the impact of MMC on target protein structure and function both in cell-free and cell-based systems.

## 8 Future directions

Incorporating crowding into *in vitro* models, both intracellularly and extracellularly has demonstrated its importance for more accurate research.

Modifying intracellular MMC through extracellular osmotic changes can be deleterious: decreased intracellular crowding induced by ionophore promotes HeLa cells swelling without affecting viability for 8 h [176], swelling of canine ischemic myocardial cells indicates necrosis [177], excessive intracellular crowding leads to cell shrinkage which precedes apoptosis and can trigger caspase activation and cytochrome *c* release from mitochondria in cells [176,38]. Although altering intracellular MMC homeostasis may be harmful, investigating these modifications remains essential, as intracellular MMC is altered in some pathologies, as it is reported in osteoarthritis [121]. Further studies should determine whether intracellular MMC is modified in other diseases, such as senescence and cancer, where it may play a role in regulating gene expression, epigenetics and cell fate.

**Fig. 6:**
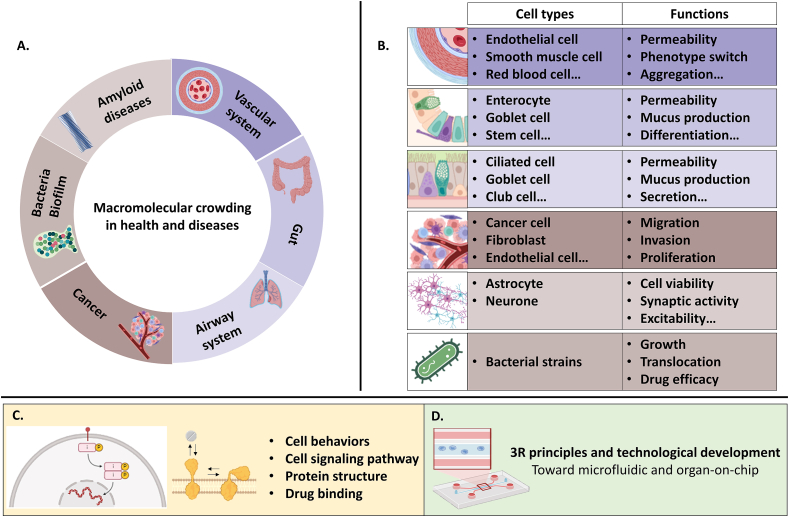
***Proposition for future directions.****Macromolecular crowding needs to be integrated toin vitro models of health and diseases: vascular system, gut, respiratory system, cancer, bacteria biofilm, amyloid diseases. This list is not exclusive. (A). Some cell types composing the different tissues and the cell behaviors that can be studied are listed (B). The different mechanisms that can be studied are cells behaviors, signaling pathways, proteins structures, and drug binding and efficacy (C). Future development consists in integrating crowding to microfluidic and organ-on-chip models which align with the ever-growing 3R principles (D). In vascular network, cells can be cultured in crowded environment to look at endothelial cell's permeability, smooth muscles cell's phenotypic switching or red blood cells aggregation. Atherosclerosis can be mimicked by adding oxidized low-density lipoproteins to the crowded environment. Gut cells (enterocytes, goblets cells or stem cells) and airway cells (ciliated, goblet or club cells) can be cultured in crowded environment to look at permeability, mucus production, or secretion. The gut and airway models can be complexified by adding bacteria to mimic infection and look at bacterial translocation across the epithelial barrier. Similarly, cancer cells, in coculture with vascular cells or not, can be cultured in crowded environment to look at their metastatic capacities. Neuronal cells can also be cultured in crowded environment to look at the impact of amyloid fibrils toxicity. All these models can be used to test drug efficacy and their target protein conformation which might be related to the efficacy of the drugs. Biorender imageswere used to generate the figure. (For interpretation of the references to colour in this figure legend, the reader is referred to the Web version of this article.)*

Regarding extracellular MMC, current research focuses on three major areas: (i) maintaining difficult-to-culture cells while preserving their phenotype and function; (ii) promoting ECM deposition for *in vitro* tissue engineering; and (iii) facilitating ECM deposition for fibrosis drug screening [94,99,113]. These research areas should be continued and expanded. Additionally, further studies should aim to model both diseases and healthy conditions *in vitro* (Fig. 6). Most cells *in vivo* exist within a naturally crowded environment, whereas in culture, they are maintained in a dilute environment [9]. It is crucial to develop healthy models that incorporate MMC to determine whether cells naturally existing in crowded environments retain their phenotype and function in both dilute and crowded conditions. These models should then be adapted to mimic pathological conditions. For example, culturing vascular cells (endothelial cells or VSMC) in crowded conditions supplemented with oxidized low-density lipoproteins, leucocytes and/or monocytes could better replicate atherosclerotic conditions to study cells phenotypic and functional switch, and to improve drug screening and clinical translation. Integrating cancer cells and/or cancer drugs into these crowded vascular wall models may provide new insights into metastasis mechanisms. Few studies have investigated the role of MMC on vascular cells, which naturally exist in a crowded environment [159,168]. However, to our knowledge, no researches explore the role of MMC in vascular pathologies, such as atherosclerosis, or the associated signaling pathways. Similarly, no studies look at tumor metastasis across vascular barrier in crowded environment. A logical next step would be to integrate MMC into organ-on-chip models to better replicate *in vivo* conditions.

Further development of tissue models is also necessary. For example, epithelial cells of the gut and respiratory systems are naturally in contact with mucus layers, which constitute crowded environments. To our knowledge, no study has examined the effects of crowding on gut or respiratory epithelial cells under healthy or disease conditions. Several diseases, such as inflammatory bowel disease and cystic fibrosis, are associated with bacterial infections and the destruction of mucus layers. Additionally, implant-related infections remain a significant health concern [178]. However, studies examining the effects of MMC on bacterial functions and development in relation to these pathologies remain scarce. Modeling crowded gut and respiratory environments by co-culturing enterocytes or ciliated cells with bacteria and/or specific drugs in the presence of crowders may provide new insights into disease mechanisms.

Developing *in vitro* cellular models that incorporate MMC for both physiological and pathological conditions is highly valuable for drug efficacy screening platforms, as well as toxicology studies of molecules such as endocrine disruptors. To fully understand the effects of drugs and toxic molecules, studies on their protein partners' structure, activity, and binding properties under crowded conditions—both in cell-free and cell-based systems must continue. These *in vitro* models of physiological and pathological conditions will become increasingly essential as biological research continues to embrace the 3Rs principle: refine, reduce, and replace animal experimentation in laboratory studies [179].

## 9 Conclusion

The effects of MMC are universal across biological systems and have significant implications for cellular physiology, molecular interactions, and overall biomolecular behavior. Intracellular MMC modulates gene expression, protein polymerization, and transport within the nucleus, cytoplasm, and organelles. Extracellular MMC enhances ECM deposition and, consequently, modulate cell behavior. MMC also regulates the polymerization of proteins implicated in neurodegenerative disorders and affects cell fate in various diseases, including osteoarthritis and cancer. One study demonstrates that MMC enhances the impermeability of capillary-like structures formed by endothelial cells in 3D *in vitro* models. Future research should focus on developing more reliable *in vitro* cell culture models and organ-on-chip systems that incorporate MMC to better emulate physiological and pathological tissues. Further MMC-based models of various tissues should be developed to explore the effects of MMC under both normal and diseases conditions, particularly in vascular, gut, and respiratory systems. These models will provide crucial insights into cellular processes, enhance our understanding of complex biological systems, and ultimately lead to more effective therapeutic strategies, improved predictive power for *in vitro* drug screening, and better translation of research findings into clinical applications.

## CRediT authorship contribution statement

**Jérémy Lagrange:** Writing – review & editing. **Gabrielle Van De Velde:** Writing – review & editing. **Patrick Lacolley:** Writing – review & editing. **Véronique Regnault:** Writing – review & editing. **Rümeyza Bascetin:** Writing – review & editing, Writing – original draft, Supervision, Project administration, Conceptualization.

## Declaration of generative AI and AI-assisted technologies in the writing process

During the preparation of this work the authors used Perplexity solely to enhance the grammatical accuracy of the manuscript.

## Declaration of competing interest

The authors declare that they have no known competing financial interests or personal relationships that could have appeared to influence the work reported in this paper.

## Data Availability

No data was used for the research described in the article.

## References

[1] Fulton A.B. (1982). How crowded is the cytoplasm?. Cell.

[2] Pizzo S.V., Lundblad R.L., Willis M.S. (2016). Composition and Function of the Interstitial Fluid, Proteolysis in the Interstitial Space.

[3] Liu X., Oh S., Kirschner M.W. (2022). The uniformity and stability of cellular mass density in mammalian cell culture. Front. Cell Dev. Biol..

[4] Pliss A., Peng X., Liu L., Kuzmin A., Wang Y., Qu J., Li Y., Prasad P.N. (2015). Single cell assay for molecular diagnostics and medicine: monitoring intracellular concentrations of macromolecules by two-photon fluorescence lifetime imaging. Theranostics.

[5] Wiśniewski J.R., Hein M.Y., Cox J., Mann M. (2014). A “proteomic ruler” for protein copy number and concentration estimation without spike-in standards. Mol. Cell. Proteomics.

[6] Okutucu B., Dinçer A., Habib Ö., Zihnioglu F. (2007). Comparison of five methods for determination of total plasma protein concentration. J. Biochem. Biophys. Methods.

[7] Bascetin R., Laurent-Issartel C., Blanc-Fournier C., Vendrely C., Kellouche S., Carreiras F., Gallet O., Leroy-Dudal J. (2021). A biomimetic model of 3D fluid extracellular macromolecular crowding microenvironment fine-tunes ovarian cancer cells dissemination phenotype. Biomaterials.

[8] Hynes R.O., Naba A. (2012). Overview of the matrisome-An inventory of extracellular matrix constituents and functions. Cold Spring Harbor Perspect. Biol..

[9] Tsiapalis D., Zeugolis D.I. (2021). It is time to crowd your cell culture media - physicochemical considerations with biological consequences. Biomaterials.

[10] Subramanya A.R., Boyd-Shiwarski C.R. (2024). Molecular crowding: physiologic sensing and control. Annu. Rev. Physiol..

[11] Zhou H.X., Rivas G., Minton A.P. (2008). Macromolecular crowding and confinement: biochemical, biophysical, and potential physiological consequences. Annu. Rev. Biophys..

[12] Jalihal A.P., Schmidt A., Gao G., Little S.R., Pitchiaya S., Walter N.G. (2021). Hyperosmotic phase separation: condensates beyond inclusions, granules and organelles. J. Biol. Chem..

[13] Minton A.P. (1992). Confinement as a determinant of macromolecular structure and reactivity. Biophys. J..

[14] Zhou H.X. (2008). Protein folding in confined and crowded environments. Arch. Biochem. Biophys..

[15] Minton A.P. (1998). Molecular crowding: analysis of effects of high concentrations of inert cosolutes on biochemical equilibria and rates in terms of volume exclusion. Methods Enzymol..

[16] Sarkar M., Li C., Pielak G.J. (2013). Soft interactions and crowding. Biophys Rev.

[17] André A.A.M., Spruijt E. (2020). Liquid–liquid phase separation in crowded environments. Int. J. Mol. Sci..

[18] Rusinga F.I., Weis D.D. (2017). Soft interactions and volume exclusion by polymeric crowders can stabilize or destabilize transient structure in disordered proteins depending on polymer concentration. Proteins: Struct., Funct., Bioinf..

[19] Assunção M., Wong C.W., Richardson J.J., Tsang R., Beyer S., Raghunath M., Blocki A. (2020). Macromolecular dextran sulfate facilitates extracellular matrix deposition by electrostatic interaction independent from a macromolecular crowding effect. Mater. Sci. Eng. C.

[20] Lareu R.R., Subramhanya K.H., Peng Y., Benny P., Chen C., Wang Z., Rajagopalan R., Raghunath M. (2007). Collagen matrix deposition is dramatically enhanced in vitro when crowded with charged macromolecules: the biological relevance of the excluded volume effect. FEBS Lett..

[21] Norred S.E., Caveney P.M., Chauhan G., Collier L.K., Collier C.P., Abel S.M., Simpson M.L. (2018). Macromolecular crowding induces spatial correlations that control gene expression bursting patterns. ACS Synth. Biol..

[22] Chapanian R., Kwan D.H., Constantinescu I., Shaikh F.A., Rossi N.A.A., Withers S.G., Kizhakkedathu J.N. (2014). Enhancement of biological reactions on cell surfaces via macromolecular crowding. Nat. Commun..

[23] Motte-Signoret E., Jlassi M., Lecoq L., Wachter P.Y., Durandy A., Boileau P. (2023). Early elevated alkaline phosphatase as a surrogate biomarker of ongoing metabolic bone disease of prematurity. Eur. J. Pediatr..

[24] Keyel P.A. (2017). Dnases in health and disease. Dev. Biol..

[25] Homchaudhuri L., Sarma N., Swaminathan R. (2006). Effect of crowding by dextrans and Ficolls on the rate of alkaline phosphatase–catalyzed hydrolysis: a size-dependent investigation. Biopolymers.

[26] Sasaki Y., Miyoshi D., Sugimoto N. (2007). Regulation of DNA nucleases by molecular crowding. Nucleic Acids Res..

[27] Kuznetsova I.M., Turoverov K.K., Uversky V.N. (2014). What macromolecular crowding can do to a protein.

[28] Poolman B. (2023). Physicochemical homeostasis in bacteria. FEMS Microbiol. Rev..

[29] Delarue M., Brittingham G.P., Pfeffer S., Surovtsev I.V., Pinglay S., Kennedy K.J., Schaffer M., Gutierrez J.I., Sang D., Poterewicz G., Chung J.K., Plitzko J.M., Groves J.T., Jacobs-Wagner C., Engel B.D., Holt L.J. (2018). mTORC1 controls phase separation and the biophysical properties of the cytoplasm by tuning crowding. Cell.

[30] Miermont A., Waharte F., Hu S., McClean M.N., Bottani S., Leon S., Hersen P. (2013). Severe osmotic compression triggers a slowdown of intracellular signaling, which can be explained by molecular crowding. Proc. Natl. Acad. Sci..

[31] Shen Y., Ori-McKenney K.M. (2023). Macromolecular crowding tailors the microtubule cytoskeleton through tubulin modifications and microtubule-associated proteins. bioRxiv.

[32] Park J., Lee M., Lee B., Castaneda N., Tetard L., Kang E.H. (2021). Crowding tunes the organization and mechanics of actin bundles formed by crosslinking proteins. FEBS Lett..

[33] Quinn S.D., Dresser L., Graham S., Conteduca D., Shepherd J., Leake M.C. (2022). Crowding-induced morphological changes in synthetic lipid vesicles determined using smFRET. Front. Bioeng. Biotechnol..

[34] Richter K., Nessling M., Lichter P. (2007). Experimental evidence for the influence of molecular crowding on nuclear architecture. J. Cell Sci..

[35] Richter K., Nessling M., Lichter P. (2008). Macromolecular crowding and its potential impact on nuclear function. Biochim. Biophys. Acta Mol. Cell Res..

[36] Bakshi S., Siryaporn A., Goulian M., Weisshaar J.C. (2012). Superresolution imaging of ribosomes and RNA polymerase in live Escherichia coli cells. Mol. Microbiol..

[37] Bulthuis E.P., Dieteren C.E.J., Bergmans J., Berkhout J., Wagenaars J.A., van de Westerlo E.M.A., Podhumljak E., Hink M.A., Hesp L.F.B., Rosa H.S., Malik A.N., Lindert M.K., Willems P.H.G.M., Gardeniers H.J.G.E., den Otter W.K., Adjobo‐Hermans M.J.W., Koopman W.J.H. (2023). Stress‐dependent macromolecular crowding in the mitochondrial matrix. EMBO J..

[38] Maeno E., Ishizaki Y., Kanaseki T., Hazama A., Okada Y. (2000). Normotonic cell shrinkage because of disordered volume regulation is an early prerequisite to apoptosis. Proc. Natl. Acad. Sci. U. S. A..

[39] André A.A.M., Yewdall N.A., Spruijt E. (2023). Crowding-induced phase separation and gelling by co-condensation of PEG in NPM1-rRNA condensates. Biophys. J..

[40] Ferrolino M.C., Mitrea D.M., Michael J.R., Kriwacki R.W. (2018). Compositional adaptability in NPM1-SURF6 scaffolding networks enabled by dynamic switching of phase separation mechanisms. Nat. Commun..

[41] Nunes P., Roth I., Meda P., Féraille E., Brown D., Hasler U. (2015). Ionic imbalance, in addition to molecular crowding, abates cytoskeletal dynamics and vesicle motility during hypertonic stress. Proc. Natl. Acad. Sci..

[42] Sood P., Murthy K., Kumar V., Nonet M.L., Menon G.I., Koushika S.P. (2018). Cargo crowding at actin-rich regions along axons causes local traffic jams. Traffic.

[43] Rashid R., Chee S.M.L., Raghunath M., Wohland T. (2015). Macromolecular crowding gives rise to microviscosity, anomalous diffusion and accelerated actin polymerization. Phys. Biol..

[44] Castaneda N., Lee M., Rivera-Jacquez H.J., Marracino R.R., Merlino T.R., Kang H. (2019). Actin filament mechanics and structure in crowded environments. J. Phys. Chem. B.

[45] Castaneda N., Feuillie C., Molinari M., Kang E.H. (2021). Actin bundle nanomechanics and organization are modulated by macromolecular crowding and electrostatic interactions. Front. Mol. Biosci..

[46] Demosthene B., Lee M., Marracino R.R., Heidings J.B., Kang E.H. (2023). Molecular basis for actin polymerization kinetics modulated by solution crowding. Biomolecules.

[47] Ge J., Bouriyaphone S.D., Serebrennikova T.A., Astashkin A.V., Nesmelov Y.E. (2016). Macromolecular crowding modulates actomyosin kinetics. Biophys. J..

[48] Sun Y.L., Ge B.Q., Li M.Z., Wang L., Chen Z.X. (2023). The effect of macromolecular crowding degree on the self-assembly of fatty acid and lipid hydrolysis. Npj Science of Food.

[49] Bancaud A., Huet S., Daigle N., Mozziconacci J., Beaudouin J., Ellenberg J. (2009). Molecular crowding affects diffusion and binding of nuclear proteins in heterochromatin and reveals the fractal organization of chromatin. EMBO J..

[50] Akabayov B., Akabayov S.R., Lee S.J., Wagner G., Richardson C.C. (2013). Impact of macromolecular crowding on DNA replication. Nat. Commun..

[51] Sun L., Fang J. (2016). Macromolecular crowding effect is critical for maintaining SIRT1's nuclear localization in cancer cells. Cell Cycle.

[52] Partikian A., Ölveczky B., Swaminathan R., Li Y., Verkman A.S. (1998). Rapid diffusion of green fluorescent protein in the mitochondrial matrix. J. Cell Biol..

[53] Brantová O., Tesařová M., Hansíková H., Elleder M., Zeman J., Sládková J. (2006). Ultrastructural changes of mitochondria in the cultivated skin fibroblasts of patients with point mutations in mitochondrial DNA. Ultrastruct. Pathol..

[54] Lofaro F.D., Boraldi F., Garcia-Fernandez M., Estrella L., Valdivielso P., Quaglino D. (2020). Relationship between mitochondrial structure and bioenergetics in pseudoxanthoma elasticum dermal fibroblasts. Front. Cell Dev. Biol..

[55] Castro-Sepulveda M., Tapia G., Tuñón-Suárez M., Diaz A., Marambio H., Valero-Breton M., Fernández-Verdejo R., Zbinden-Foncea H. (2022). Severe COVID-19 correlates with lower mitochondrial cristae density in PBMCs and greater sitting time in humans. Phys. Rep..

[56] Alberti S., Gladfelter A., Mittag T. (2019). Considerations and challenges in studying liquid-liquid phase separation and biomolecular condensates. Cell.

[57] Miyagi T., Yamanaka Y., Harada Y., Narumi S., Hayamizu Y., Kuroda M., Kanekura K. (2021). An improved macromolecular crowding sensor CRONOS for detection of crowding changes in membrane-less organelles under stressed conditions. Biochem. Biophys. Res. Commun..

[58] Lafontaine D.L.J., Riback J.A., Bascetin R., Brangwynne C.P. (2020). The nucleolus as a multiphase liquid condensate. Nat. Rev. Mol. Cell Biol..

[59] Bounedjah O., Hamon L., Savarin P., Desforges B., Curmi P.A., Pastré D. (2012). Macromolecular crowding regulates assembly of mRNA stress granules after osmotic stress: new role for compatible osmolytes. J. Biol. Chem..

[60] Mathur A., Ghosh R., Nunes-Alves A. (2024). Recent progress in modeling and simulation of biomolecular crowding and condensation inside cells. J. Chem. Inf. Model..

[61] Larhed A.W., Artursson P., Björk E. (1998). The influence of intestinal mucus components on the diffusion of drugs. Pharm. Res..

[62] Hsieh W.Y., Chen M.W., Ho H.T., You T.M., Lu Y.T. (2006). Identification of differentially expressed proteins in human malignant pleural effusions. Eur. Respir. J..

[63] Berlyne G.M., Li J., Kwan T., Caruso C. (1989). Oedema protein concentrations for differentiation of cellulitis and deep vein thrombosis. Lancet.

[64] McLenachan S., Hao E., Zhang D., Zhang L., Edel M., Chen F. (2017). Bioengineered Bruch’s-like extracellular matrix promotes retinal pigment epithelial differentiation. Biochem Biophys Rep.

[65] Bissell M.J., Hall H.G., Parry G. (1982). How does the extracellular matrix direct gene expression?. J. Theor. Biol..

[66] Satyam A., Kumar P., Fan X., Gorelov A., Rochev Y., Joshi L., Peinado H., Lyden D., Thomas B., Rodriguez B., Raghunath M., Pandit A., Zeugolis D. (2014). Macromolecular crowding meets tissue engineering by self-assembly: a paradigm shift in regenerative medicine. Adv. Mater..

[67] Satyam A., Kumar P., Cigognini D., Pandit A., Zeugolis D.I. (2016). Low, but not too low, oxygen tension and macromolecular crowding accelerate extracellular matrix deposition in human dermal fibroblast culture. Acta Biomater..

[68] Cigognini D., Gaspar D., Kumar P., Satyam A., Alagesan S., Sanz-Nogués C., Griffin M., O'Brien T., Pandit A., Zeugolis D.I. (2016). Macromolecular crowding meets oxygen tension in human mesenchymal stem cell culture - a step closer to physiologically relevant in vitro organogenesis. Sci. Rep..

[69] Zeiger A.S., Loe F.C., Li R., Raghunath M., van Vliet K.J. (2012). Macromolecular crowding directs extracellular matrix organization and mesenchymal stem cell behavior. PLoS One.

[70] Shahid S., Hassan M.I., Islam A., Ahmad F. (2017). Size-dependent studies of macromolecular crowding on the thermodynamic stability, structure and functional activity of proteins: in vitro and in silico approaches. Biochim. Biophys. Acta Gen. Subj..

[71] Kipps E., Tan D.S.P., Kaye S.B. (2013). Meeting the challenge of ascites in ovarian cancer: new avenues for therapy and research. Nat. Rev. Cancer.

[72] Matte I., Lane D., Laplante C., Rancourt C., Piché A. (2012). Profiling of cytokines in human epithelial ovarian cancer ascites. Am. J. Cancer Res..

[73] Puiffe M.L., Le Page C., Filali-Mouhim A., Zietarska M., Ouellet V., Tonin P.N., Chevrette M., Provencher D.M., Mes-Masson A.M. (2007). Characterization of ovarian cancer ascites on cell invasion, proliferation, spheroid formation, and gene expression in an in vitro model of epithelial ovarian cancer. Neoplasia.

[74] Harve K.S., Raghunath M., Lareu R.R., Rajagopalan R. (2006). Macromolecular crowding in biological systems: dynamic light scattering (DLS) to quantify the excluded volume effect (EVE). Biophys. Rev. Lett..

[75] Leduc M., Guay D., Leask R.L., Coulombe S. (2009). Cell permeabilization using a non-thermal plasma. New J. Phys..

[76] Lopez-Sanchez P., Schuster E., Wang D., Gidley M.J., Strom A. (2015). Diffusion of macromolecules in self-assembled cellulose/hemicellulose hydrogels. Soft Matter.

[77] Ranganathan V.T., Bazmi S., Wallin S., Liu Y., Yethiraj A. (2022). Is Ficoll a colloid or polymer? A multitechnique study of a prototypical excluded-volume macromolecular crowder. Macromolecules.

[78] Rashid R., Lim N.S.J., Chee S.M.L., Png S.N., Wohland T., Raghunath M. (2014). Novel use for Polyvinylpyrrolidone as a macromolecular crowder for enhanced extracellular matrix deposition and cell proliferation. Tissue Eng. C Methods.

[79] Doubková M., Knitlová J., Vondrášek D., Eckhardt A., Novotný T., Ošt’ádal M., Filová E., Bačáková L. (2024). Harnessing the biomimetic effect of macromolecular crowding in the cell-derived model of clubfoot fibrosis. Biomacromolecules.

[80] Ranamukhaarachchi S.K., Modi R.N., Han A., Velez D.O., Kumar A., Engler A.J., Fraley S.I. (2019). Macromolecular crowding tunes 3D collagen architecture and cell morphogenesis. Biomater. Sci..

[81] Titus A.R., Madeira P.P., Ferreira L.A., Chernyak V.Y., Uversky V.N., Zaslavsky B.Y. (2022). Mechanism of phase separation in aqueous two-phase systems. Int. J. Mol. Sci..

[82] Armstrong J.K., Wenby R.B., Meiselman H.J., Fisher T.C. (2004). The hydrodynamic radii of macromolecules and their effect on red blood cell aggregation. Biophys. J..

[83] Parray Z.A., Ahmad F., Chaudhary A.A., Rudayni H.A., Al-Zharani M., Hassan M.I., Islam A. (2022). Size-dependent interplay of volume exclusion versus soft interactions: cytochrome c in macromolecular crowded environment. Front. Mol. Biosci..

[84] Stewart C.J., Olgenblum G.I., Propst A., Harries D., Pielak G.J. (2023). Resolving the enthalpy of protein stabilization by macromolecular crowding. Protein Sci..

[85] Mardoum W.M., Gorczyca S.M., Regan K.E., Wu T.C., Robertson-Anderson R.M. (2018). Crowding induces entropically-driven changes to DNA dynamics that depend on crowder structure and ionic conditions. Front. Physiol..

[86] Biswas S., Kundu J., Mukherjee S.K., Chowdhury P.K. (2018). Mixed macromolecular crowding: a protein and solvent perspective. ACS Omega.

[87] Guillaumin S., Gurdal M., Zeugolis D.I. (2024). Gums as macromolecular crowding agents in human skin fibroblast cultures. Life.

[88] Yoo Y.I., Ko K.W., Cha S.G., Park S.Y., Woo J., Han D.K. (2022). Highly effective induction of cell-derived extracellular matrix by macromolecular crowding for osteogenic differentiation of mesenchymal stem cells. J. Ind. Eng. Chem..

[89] Finn T.E., Nunez A.C., Sunde M., Easterbrook-Smith S.B. (2012). Serum albumin prevents protein aggregation and amyloid formation and retains chaperone-like activity in the presence of physiological ligands. J. Biol. Chem..

[90] Seeliger J., Werkmüller A., Winter R. (2013). Macromolecular crowding as a suppressor of human IAPP fibril formation and cytotoxicity. PLoS One.

[91] Garnica-Galvez S., Korntner S.H., Skoufos I., Tzora A., Diakakis N., Prassinos N., Zeugolis D.I. (2021). Hyaluronic acid as macromolecular crowder in equine adipose-derived stem cell cultures. Cells.

[92] Dewavrin J.-Y., Hamzavi N., Shim V.P.W., Raghunath M. (2014). Tuning the architecture of three-dimensional collagen hydrogels by physiological macromolecular crowding. Acta Biomater..

[93] Guan S., Wu S., Li G., Xiao J., Gao B. (2023). Macromolecular crowding facilitates rapid fabrication of intact, robust cell sheets. Biotechnol. Lett..

[94] Lareu R.R., Arsianti I., Subramhanya H.K., Yanxian P., Raghunath M. (2007). In vitro enhancement of collagen matrix formation and crosslinking for applications in tissue engineering: a preliminary study. Tissue Eng..

[95] Kumar P., Satyam A., Fan X., Collin E., Rochev Y., Rodriguez B.J., Gorelov A., Dillon S., Joshi L., Raghunath M., Pandit A., Zeugolis D.I. (2015). Macromolecularly crowded in vitro microenvironments accelerate the production of extracellular matrix-rich supramolecular assemblies. Sci. Rep..

[96] Djalali-Cuevas A., Rettel M., Stein F., Savitski M., Kearns S., Kelly J., Biggs M., Skoufos I., Tzora A., Prassinos N., Diakakis N., Zeugolis D.I. (2024). Macromolecular crowding in human tenocyte and skin fibroblast cultures: a comparative analysis. Mater Today Bio.

[97] Rampin A., Rossoni A., Chaniotaki L., Gkiatas I.S., Tzora A., Skoufos I., Diakakis N., Prassinos N., Zeugolis D.I. (2024). Xenogeneic versus allogeneic serum and macromolecular crowding in human tenocyte cultures. Eur. J. Cell Biol..

[98] Chen C.Z.C., Peng Y.X., Wang Z.B., V Fish P., Kaar J.L., Koepsel R.R., Russell A.J., Lareu R.R., Raghunath M. (2009). The Scar-in-a-Jar: studying potential antifibrotic compounds from the epigenetic to extracellular level in a single well. Br. J. Pharmacol..

[99] Coentro J.Q., May U., Prince S., Zwaagstra J., Ritvos O., Järvinen T.A.H., Zeugolis D.I. (2021). Adapting the scar-in-a-jar to skin fibrosis and screening traditional and contemporary anti-fibrotic therapies. Front. Bioeng. Biotechnol..

[100] Puerta Cavanzo N., Bigaeva E., Boersema M., Olinga P., Bank R.A. (2021). Macromolecular crowding as a tool to screen anti-fibrotic drugs: the scar-in-a-jar system revisited. Front. Med..

[101] Fan C., Lim L.K.P., Loh S.Q., Ying Lim K.Y., Upton Z., Leavesley D. (2019). Application of “macromolecular crowding” in vitro to investigate the naphthoquinones shikonin, naphthazarin and related analogues for the treatment of dermal scars. Chem. Biol. Interact..

[102] Graham J., Raghunath M., Vogel V. (2019). Fibrillar fibronectin plays a key role as nucleator of collagen I polymerization during macromolecular crowding-enhanced matrix assembly. Biomater. Sci..

[103] Satz-Jacobowitz B., Taye N., Karoulias S.Z., Hubmacher D. (2022). Macromolecular crowding enhances fibrillin-1 deposition in the extracellular matrix. Eur. Cell. Mater..

[104] Ramalingam R., Jiang G., Larjava H., Häkkinen L. (2023). Macromolecular crowding regulates matrix composition and gene expression in human gingival fibroblast cultures. Sci. Rep..

[105] Benny P., Badowski C., Lane E.B., Raghunath M. (2015). Making more matrix: enhancing the deposition of dermal-epidermal junction components in vitro and accelerating organotypic skin culture development, using macromolecular crowding. Tissue Eng Part A.

[106] Valli M., Rossi A., Forlino A., Tenni R., Cetta G. (1993). Extracellular matrix deposition in cultured dermal fibroblasts from four probands affected by osteogenesis imperfecta. Matrix.

[107] Raghunath M., Superti-Furga A., Godfrey M., Steinmann B. (1993). Decreased extracellular deposition of fibrillin and decorin in neonatal Marfan syndrome fibroblasts. Hum. Genet..

[108] Godfrey M., Raghunath M., Cisler J., Bevins C.L., DePaepe A., Di Rocco M., Gregoritch J., Imaizumi K., Kaplan P., Kuroki Y., Silberbach M., Superti-Furga A., Van Thienen M.N., Vetter U., Steinmann B. (1995). Abnormal morphology of fibrillin microfibrils in fibroblast cultures from patients with neonatal marfan syndrome. Am. J. Pathol..

[109] Peng Y., Bocker M.T., Holm J., Toh W.S., Hughes C.S., Kidwai F., Lajoie G.A., Cao T., Lyko F., Raghunath M. (2012). Human fibroblast matrices bio-assembled under macromolecular crowding support stable propagation of human embryonic stem cells. J Tissue Eng Regen Med.

[110] Ang X.M., Lee M.H.C., Blocki A., Chen C., Ong L.L.S., Asada H.H., Sheppard A., Raghunath M. (2013). Macromolecular crowding amplifies adipogenesis of human bone marrow-derived mesenchymal stem cells by enhancing the pro-adipogenic microenvironment. Tissue Eng Part A.

[111] Korntner S.H., Di Nubila A., Gaspar D., Zeugolis D.I. (2023). Macromolecular crowding in animal component-free, xeno-free and foetal bovine serum media for human bone marrow mesenchymal stromal cell expansion and differentiation. Front. Bioeng. Biotechnol..

[112] Prewitz M.C., Stißel A., Friedrichs J., Träber N., Vogler S., Bornhäuser M., Werner C. (2015). Extracellular matrix deposition of bone marrow stroma enhanced by macromolecular crowding. Biomaterials.

[113] Satyam A., Tsokos M.G., Tresback J.S., Zeugolis D.I., Tsokos G.C. (2020). Cell-derived extracellular matrix-rich biomimetic substrate supports podocyte proliferation, differentiation, and maintenance of native phenotype. Adv. Funct. Mater..

[114] Vo A.N., Kundu S., Strong C., Jung O., Lee E., Song M.J., Boutin M.E., Raghunath M., Ferrer M. (2022). Enhancement of neuroglial extracellular matrix formation and physiological activity of dopaminergic neural cocultures by macromolecular crowding. Cells.

[115] Chiejina M., Kudaravalli P., Samant H. (2023). Ascites. StatPearls.

[116] Paricaud K., Moulis G., Combis M.S., Sailler L., Arlet P. (2014). Causes of protidemia above 100 g/L. Eur. J. Intern. Med..

[117] Wang Q.R., Long J., Wang C.C., Hu J.L., Lin N., Tang S.H. (2023). Case report of atypical undernutrition of hypoproteinemia type. Open Life Sci..

[118] Mangialardi R.J., Martin G.S., Bernard G.R., Wheeler A.P., Christman B.W., Dupont W.D., Higgins S.B., Swindell B.B. (2000). Hypoproteinemia predicts acute respiratory distress syndrome development, weight gain, and death in patients with sepsis. Ibuprofen in Sepsis Study Group. Crit. Care Med..

[119] Shanfield S., Campbell P., Baumgarten M., Bloebaum R., Sarmiento A. (1988). Synovial fluid osmolality in osteoarthritis and rheumatoid arthritis. Clin. Orthop. Relat. Res..

[120] Fujimura K., Segami N., Yoshitake Y., Tsuruoka N., Kaneyama K., Sato J., Kobayashi S. (2006). Electrophoretic separation of the synovial fluid proteins in patients with temporomandibular joint disorders. Oral Surg. Oral Med. Oral Pathol. Oral Radiol. Endod..

[121] Govindaraj K., Meteling M., van Rooij J., Becker M., van Wijnen A.J., van den Beucken J.J.J.P., Ramos Y.F.M., van Meurs J., Post J.N., Leijten J. (2024). Osmolarity-induced altered intracellular molecular crowding drives osteoarthritis pathology. Adv. Sci..

[122] Burkewitz K., Choe K., Strange K. (2011). Hypertonic stress induces rapid and widespread protein damage in C. elegans. Am. J. Physiol. Cell Physiol..

[123] Patel S.P., Nikam T., Sreepathi B., Karankar V.S., Jaiswal A., Vardhan S.V., Rana A., Toga V., Srivastava N., Saraf S.A., Awasthi S. (2024). Unraveling the molecular jam: how crowding shapes protein aggregation in neurodegenerative disorders. ACS Chem. Biol..

[124] Fuertes M.A., López Mateos D., Valiente L., Rodríguez Huete A., Valbuena A., Mateu M.G. (2023). Electrostatic screening, acidic pH and macromolecular crowding increase the self-assembly efficiency of the minute virus of mice capsid in vitro. Viruses.

[125] Hatters D.M., Minton A.P., Howlett G.J. (2002). Macromolecular crowding accelerates amyloid formation by human apolipoprotein C-II. J. Biol. Chem..

[126] Siddiqui G.A., Naeem A. (2018). Aggregation of globular protein as a consequences of macromolecular crowding: a time and concentration dependent study. Int. J. Biol. Macromol..

[127] Dewavrin J.Y., Abdurrahiem M., Blocki A., Musib M., Piazza F., Raghunath M. (2015). Synergistic rate boosting of collagen fibrillogenesis in heterogeneous mixtures of crowding agents. J. Phys. Chem. B.

[128] Abedini A., Schmidt A.M. (2013). Mechanisms of islet amyloidosis toxicity in type 2 diabetes. FEBS Lett..

[129] Berwick R., Vaux D.J., Jean L. (2018). Multiphasic effect of vinyl pyrrolidone polymers on amyloidogenesis, from macromolecular crowding to inhibition. Biochem. J..

[130] Longhena F., Faustini G., Missale C., Pizzi M., Spano P., Bellucci A. (2017). The contribution of α-synuclein spreading to Parkinson's disease synaptopathy. Neural Plast..

[131] Horvath I., Kumar R., Wittung-Stafshede P. (2021). Macromolecular crowding modulates α-synuclein amyloid fiber growth. Biophys. J..

[132] Uversky V.N., Cooper E.M., Bower K.S., Li J., Fink A.L. (2002). Accelerated α-synuclein fibrillation in crowded milieu. FEBS Lett..

[133] Bjorkli C., Sandvig A., Sandvig I. (2020). Bridging the gap between fluid biomarkers for Alzheimer’s disease, model systems, and patients. Front. Aging Neurosci..

[134] Lee C.F., Bird S., Shaw M., Jean L., Vaux D.J. (2012). Combined effects of agitation, macromolecular crowding, and interfaces on amyloidogenesis. J. Biol. Chem..

[135] Wu Y., Teng N., Li S. (2016). Effects of macromolecular crowding and osmolyte on human Tau fibrillation. Int. J. Biol. Macromol..

[136] Zhou Z., Fan J.B., Zhu H.L., Shewmaker F., Yan X., Chen X., Chen J., Xiao G.F., Guo L., Liang Y. (2009). Crowded cell-like environment accelerates the nucleation step of amyloidogenic protein misfolding. J. Biol. Chem..

[137] Ma Q., Fan J.B., Zhou Z., Zhou B.R., Meng S.R., Hu J.Y., Chen J., Liang Y. (2012). The contrasting effect of macromolecular crowding on amyloid fibril formation. PLoS One.

[138] Legleiter J., Mitchell E., Lotz G.P., Sapp E., Ng C., DiFiglia M., Thompson L.M., Muchowski P.J. (2010). Mutant huntingtin fragments form Oligomers in a polyglutamine length-dependent manner in vitro and in vivo. J. Biol. Chem..

[139] Groover S.E., Adegbuyiro A., Fan C.K., Hodges B.L., Beasley M., Taylor K., Stonebraker A.R., Siriwardhana C., Legleiter J. (2021). Macromolecular crowding in solution alters huntingtin interaction and aggregation at interfaces. Colloids Surf. B Biointerfaces.

[140] Scheckel C., Aguzzi A. (2018). Prions, prionoids and protein misfolding disorders. Nat. Rev. Genet..

[141] Zhou Z., Yan X., Pan K., Chen J., Xie Z.S., Xiao G.F., Yang F.Q., Liang Y. (2011). Fibril formation of the rabbit/human/bovine prion proteins. Biophys. J..

[142] Mittal S., Chowhan R.K., Singh L.R. (2015). Macromolecular crowding: macromolecules friend or foe. Biochim. Biophys. Acta Gen. Subj..

[143] Ma Q., Hu J.Y., Chen J., Liang Y. (2013). The role of crowded physiological environments in prion and prion-like protein aggregation. Int. J. Mol. Sci..

[144] Fang J., Wang H., Zhou Y., Zhang H., Zhou H., Zhang X. (2021). Slimy partners: the mucus barrier and gut microbiome in ulcerative colitis. Exp. Mol. Med..

[145] Arias S.L., Brito I.L. (2021). Biophysical determinants of biofilm formation in the gut. Curr Opin Biomed Eng.

[146] De Weirdt R., Van De Wiele T. (2015). Micromanagement in the gut: microenvironmental factors govern colon mucosal biofilm structure and functionality. Npj Biofilms and Microbiomes.

[147] Hall-Stoodley L., McCoy K.S. (2022). Biofilm aggregates and the host airway-microbial interface. Front. Cell. Infect. Microbiol..

[148] Kolpen M., Kragh K.N., Enciso J.B., Faurholt-Jepsen D., Lindegaard B., Egelund G.B., Jensen A.V., Ravn P., Mathiesen I.H.M., Gheorge A.G., Hertz F.B., Qvist T., Whiteley M., Jensen P.Ø., Bjarnsholt T. (2022). Bacterial biofilms predominate in both acute and chronic human lung infections. Thorax.

[149] Ghosh P., Mondal J., Ben-Jacob E., Levine H. (2015). Mechanically-driven phase separation in a growing bacterial colony. Proc. Natl. Acad. Sci. U. S. A..

[150] Zhao A., Sun J., Liu Y. (2023). Understanding bacterial biofilms: from definition to treatment strategies. Front. Cell. Infect. Microbiol..

[151] Pittas T., Zuo W., Boersma A.J. (2023). Cell wall damage increases macromolecular crowding effects in the Escherichia coli cytoplasm. iScience.

[152] Siri M., Herrera M., Moyano A.J., Celej M.S. (2021). Influence of the macromolecular crowder alginate in the fibrillar organization of the functional amyloid FapC from Pseudomonas aeruginosa. Arch. Biochem. Biophys..

[153] Angeles-Martinez L., Hatzimanikatis V. (2021). The influence of the crowding assumptions in biofilm simulations. PLoS Comput. Biol..

[154] Uruén C., Chopo-Escuin G., Tommassen J., Mainar-Jaime R.C., Arenas J. (2020). Biofilms as promoters of bacterial antibiotic resistance and tolerance. Antibiotics.

[155] Kühn M.J., Schmidt F.K., Eckhardt B., Thormann K.M. (2017). Bacteria exploit a polymorphic instability of the flagellar filament to escape from traps. Proc. Natl. Acad. Sci. U. S. A..

[156] Biswas P., Roy P., Jana S., Ray D., Das J., Chaudhuri B., Basunia R.R., Sinha B., Sinha D.K. (2024). Exploring the role of macromolecular crowding and TNFR1 in cell volume control. Elife.

[157] Berger J.M., Preusser M., Berghoff A.S., Bergen E.S. (2023). Malignant ascites: current therapy options and treatment prospects. Cancer Treat Rev..

[158] Tamsma J.T., Keizer H.J., Meinders A.E. (2001). Pathogenesis of malignant ascites: starling's law of capillary hemodynamics revisited. Ann. Oncol..

[159] Gonzalez-Molina J., Mendonça da Silva J., Fuller B., Selden C. (2019). The extracellular fluid macromolecular composition differentially affects cell-substrate adhesion and cell morphology. Sci. Rep..

[160] Winter A., Salamonsen L.A., Evans J. (2020). Modelling fibroid pathology: development and manipulation of a myometrial smooth muscle cell macromolecular crowding model to alter extracellular matrix deposition. Mol. Hum. Reprod..

[161] Shologu N., Gurdal M., Szegezdi E., FitzGerald U., Zeugolis D.I. (2022). Macromolecular crowding in the development of a three-dimensional organotypic human breast cancer model. Biomaterials.

[162] Aarts P.A.M.M., Van den Broek S.A.T., Prins G.W., Kuiken G.D.C., Sixma J.J., Heethaar R.M. (1988). Blood platelets are concentrated near the wall and red blood cells, in the center in flowing blood. Arteriosclerosis: An Official Journal of the American Heart Association, Inc..

[163] Wong K.H.K., Sandlin R.D., Carey T.R., Miller K.L., Shank A.T., Oklu R., Maheswaran S., Haber D.A., Irimia D., Stott S.L., Toner M. (2016). The role of physical stabilization in whole blood preservation. Sci. Rep..

[164] Neu B., Wenby R., Meiselman H.J. (2008). Effects of dextran molecular weight on red blood cell aggregation. Biophys. J..

[165] Qiu Y., Brown A.C., Myers D.R., Sakurai Y., Mannino R.G., Tran R., Ahn B., Hardy E.T., Kee M.F., Kumar S., Bao G., Barker T.H., Lam W.A. (2014). Platelet mechanosensing of substrate stiffness during clot formation mediates adhesion, spreading, and activation. Proc. Natl. Acad. Sci. U. S. A..

[166] Ley K., Laudanna C., Cybulsky M.I., Nourshargh S. (2007). Getting to the site of inflammation: the leukocyte adhesion cascade updated. Nat. Rev. Immunol..

[167] Imhof B.A., Aurrand-Lions M. (2004). Adhesion mechanisms regulating the migration of monocytes. Nat. Rev. Immunol..

[168] Wan H.Y., Chen J.C.H., Xiao Q., Wong C.W., Yang B., Cao B., Tuan R.S., Nilsson S.K., Ho Y.P., Raghunath M., Kamm R.D., Blocki A. (2023). Stabilization and improved functionality of three-dimensional perfusable microvascular networks in microfluidic devices under macromolecular crowding. Biomater. Res..

[169] Guan X., Hu Y., Hao J., Lu M., Zhang Z., Hu W., Li D., Li C. (2024). Stress, vascular smooth muscle cell phenotype and atherosclerosis: novel insight into smooth muscle cell phenotypic transition in atherosclerosis. Curr. Atheroscler. Rep..

[170] Liu Q., Jiang H.J., Di Wu Y., Li J.D., Sun X.H., Xiao C., Xu J.Y., Lin Z.Y. (2024). Carrageenan maintains the contractile phenotype of vascular smooth muscle cells by increasing macromolecular crowding in vitro. Eur. J. Med. Res..

[171] Dey D., Nunes-Alves A., Wade R.C., Schreiber G. (2022). Diffusion of small molecule drugs is affected by surface interactions and crowder proteins. iScience.

[172] Hassan M.M., Martin A.D., Thordarson P. (2015). Macromolecular crowding and hydrophobic effects on Fmoc-diphenylalanine hydrogel formation in PEG : water mixtures. J. Mater. Chem. B.

[173] Kasahara K., Re S., Nawrocki G., Oshima H., Mishima-Tsumagari C., Miyata-Yabuki Y., Kukimoto-Niino M., Yu I., Shirouzu M., Feig M., Sugita Y. (2021). Reduced efficacy of a Src kinase inhibitor in crowded protein solution. Nat. Commun..

[174] Good R.B., Eley J.D., Gower E., Butt G., Blanchard A.D., Fisher A.J., Nanthakumar C.B. (2019). A high content, phenotypic “scar-in-a-jar” assay for rapid quantification of collagen fibrillogenesis using disease-derived pulmonary fibroblasts. BMC Biomed Eng.

[175] Rønnow S.R., Dabbagh R.Q., Genovese F., Nanthakumar C.B., Barrett V.J., Good R.B., Brockbank S., Cruwys S., Jessen H., Sorensen G.L., Karsdal M.A., Leeming D.J., Sand J.M.B. (2020). Prolonged Scar-in-a-Jar: an in vitro screening tool for anti-fibrotic therapies using biomarkers of extracellular matrix synthesis. Respir. Res..

[176] Rana P.S., Kurokawa M., Model M.A. (2020). Evidence for macromolecular crowding as a direct apoptotic stimulus. J. Cell Sci..

[177] DiBona D., Powell W.J. (1980). Quantitative correlation between cell swelling and necrosis in myocardial ischemia in dogs. Circ. Res..

[178] Van Epps J.S., Younger J.G. (2016). Implantable device related infection. Shock.

[179] Lewis D.I. (2019). Animal experimentation: implementation and application of the 3Rs. Emerg Top Life Sci.

